# Effects of Flexible Conjugation-Break Spacers of Non-Conjugated Polymer Acceptors on Photovoltaic and Mechanical Properties of All-Polymer Solar Cells

**DOI:** 10.1007/s40820-022-00884-8

**Published:** 2022-08-13

**Authors:** Qiaonan Chen, Yung Hee Han, Leandro R. Franco, Cleber F. N. Marchiori, Zewdneh Genene, C. Moyses Araujo, Jin-Woo Lee, Tan Ngoc-Lan Phan, Jingnan Wu, Donghong Yu, Dong Jun Kim, Taek-Soo Kim, Lintao Hou, Bumjoon J. Kim, Ergang Wang

**Affiliations:** 1 Siyuan Laboratory, Guangzhou Key Laboratory of Vacuum Coating Technologies and New Energy Materials, Department of Physics, Jinan University, Guangzhou, 510632 People’s Republic of China; 2 Department of Chemistry and Chemical Engineering, Chalmers University of Technology, SE-412 96, Göteborg, Sweden; 3 Department of Chemical and Biomolecular Engineering, Korea Advanced Institute of Science and Technology (KAIST), Daejeon, 34141 Republic of Korea; 4 Department of Engineering and Physics, Karlstad University, 65188 Karlstad, Sweden; 5 Department of Chemistry and Bioscience, Aalborg University, 9220 Aalborg, Denmark; 6 Department of Mechanical Engineering, Korea Advanced Institute of Science and Technology (KAIST), Daejeon, 34141 Republic of Korea; 7 Materials Theory Division, Department of Physics and Astronomy, Uppsala University, 75120 Uppsala, Sweden; 8 Sino-Danish Center for Education and Research, 8000 Aarhus, Denmark; 9 School of Materials Science and Engineering, Zhengzhou University, Zhengzhou, 450001 People’s Republic of China

**Keywords:** All-polymer solar cells, Flexible conjugation-break spacers, Mechanical robustness, Polymer acceptors, Stretchability

## Abstract

**Highlights:**

- A series of non-conjugated acceptor polymers with flexible conjugation-break spacers (FCBSs) of different lengths were synthesized.
- The effect of FCBSs length on solubility of the acceptor polymers, and their photovoltaic and mechanical properties in all-polymer solar cells were explored.
- This work provides useful guidelines for the design of semiconducting polymers by introducing FCBS with proper length, which can giantly improved properties that are not possible to be achieved by the state-of-the-art fully conjugated polymers.

**Abstract:**

All-polymer solar cells (all-PSCs) possess attractive merits including superior thermal stability and mechanical flexibility for large-area roll-to-roll processing. Introducing flexible conjugation-break spacers (FCBSs) into backbones of polymer donor (*P*_D_) or polymer acceptor (*P*_A_) has been demonstrated as an efficient approach to enhance both the photovoltaic (PV) and mechanical properties of the all-PSCs. However, length dependency of FCBS on certain all-PSC related properties has not been systematically explored. In this regard, we report a series of new non-conjugated *P*_A_s by incorporating FCBS with various lengths (2, 4, and 8 carbon atoms in thioalkyl segments). Unlike common studies on so-called side-chain engineering, where longer side chains would lead to better solubility of those resulting polymers, in this work, we observe that the solubilities and the resulting photovoltaic/mechanical properties are optimized by a proper FCBS length (*i.e.*, C2) in *P*_A_ named PYTS-C2. Its all-PSC achieves a high efficiency of 11.37%, and excellent mechanical robustness with a crack onset strain of 12.39%, significantly superior to those of the other *P*_A_s. These results firstly demonstrate the effects of FCBS lengths on the PV performance and mechanical properties of the all-PSCs, providing an effective strategy to fine-tune the structures of *P*_A_s for highly efficient and mechanically robust PSCs.
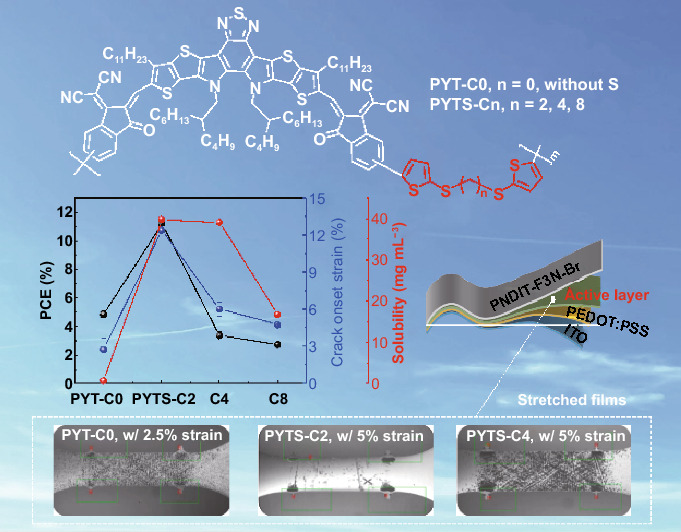

**Supplementary Information:**

The online version contains supplementary material available at 10.1007/s40820-022-00884-8.

## Introduction

Polymer solar cells (PSCs) have attracted extensive research attention due to their straight advantages of light-weight, flexibility, transparency, low cost, and facile large-area fabrication [1–5]. Driven by those benefits, non-fullerene PSCs based on tailor-made polymer donors (*P*_D_s) and continually developing fused-ring small molecular acceptors (SMAs) have boosted their power conversion efficiencies (PCEs) exceeding 18% [1, 6–19]. Aside from SMA-based ones, the pioneer work on firstly polymerized small-molecular acceptors (PSMAs) via polymerization of a large π-fused SMA building block (IDIC16) with a thiophene unit, namely PZ1, was reported by Zhang et al*.* in 2017, where PZ1 exhibits a low bandgap and high absorption coefficient, resulting in a relatively high PCE in the PSCs [3, 20]. After that, all polymer solar cells (all-PSCs) comprising *P*_D_ and PSMAs in the active layers have quickly achieved PCEs of over 16%, which show the unique merits of superior morphological stabilities of devices under thermal or mechanical stress and good compatibility with large-scale manufactures such as roll-to-roll printing [2–5, 20–33]. Nevertheless, the presence of rigid backbones in such fully conjugated polymers significantly decreases their solubility, and induces strong aggregation of polymers in the solid states, often leading to unoptimized morphology and unsatisfactory mechanical properties of the resulting active layers. It has been demonstrated that the introduction of flexible conjugation-break spacers (FCBSs) (*i.e.*, aliphatic segments) into a rigid *P*_D_ or polymer acceptor (*P*_A_) skeleton is an effective strategy for alleviating their backbone rigidity and thus significantly enhancing the solubility of generating polymers and mechanical properties of the resulting PSCs [34–39].

Recently, our group has pioneered two non-fully conjugated PSMAs, PF1-TS4 and PFY-2TS, by copolymerizing fixed lengths of FCBSs with advanced SMAs, IDIC16 and Y6 derivatives to obtain PCEs of 8.6% and 12.3%, respectively, with enhanced thermal stabilities in their all-PSCs [38–40]. In addition, the introduction of FCBSs into polymer backbones for realizing the equilibrium between PCE and mechanical properties has been reported. For example, Kim et al*.* reported a high PCE of 17% and a crack onset strain (COS) of 12% in the PSCs based on the *P*_D_ containing the FCBS unit [35]. Furthermore, our group reported a series of *P*_A_s by including different contents of FCBS, demonstrating that an all-PSC based on PYTS-0.3 (incorporating 30% FCBS, as *P*_A_) and PBDB-T (as *P*_D_) realized high photovoltaic (PV) performance with its PCE of 14.7% and robust mechanical properties with COS of 21.6% [34]. These results revealed that the incorporation of appropriate FCBSs in copolymers (either *P*_D_s or *P*_A_s) could significantly enhance solution processability, thermal stability, and mechanical ductility of PSCs, while maintaining their satisfied PV performance. However, the selection of FCBS lengths in those systems has been in a mode of “trial-and-error”, without exploring any systematic studies on impacts of FCBS lengths in *P*_A_s on their PV- and mechanical-properties of PSCs, although some polymers with different lengths of FCBSs have been employed in the field of organic field-effect transistors (OFETs) [37, 41–45].

In this work, we develop a series of non-conjugated *P*_A_s, PYT(S)-Cn, polymerized from a conjugated Y5 derivative (YBO-Br) and bis(trimethylstannyl)-bithiophene with different thio-aliphatic lengths as FCBSs between two thiophene units (TS-Cn, n stands for 0 (as a control-polymer without C-S bonds either), 2, 4, and 8 carbon atoms), through simple synthetic routes (Scheme S1). The introduction of S atom is mainly for the ease of the synthesis of FCBSs and moreover, sulfur has a balanced weak electron donating nature and moderate π-electron accepting capability into its 3d-orbitals when connected to aryl groups, which may be able to tune the highest occupied molecular orbital (HOMO) levels of the resulting polymers [46]. The incorporation of FCBSs with different lengths is found to considerably affect the solubility and molecular flexibility of the resulting *P*_A_s. Interestingly, with the increase of FCBS lengths, *P*_A_s show enhanced inter/intramolecular packings with higher crystallinity, decreased solubility and miscibility with *P*_D_. As a result, all-PSCs based on PYTS-C2 incorporated the shortest length of FCBS (2 carbon atoms) achieve a high PCE_avg_ of 11.20% (PCE_max_ of 11.37%), which significantly outperforms those of PYTS-C4 (PCE_avg_ = 3.37%) and PYTS-C8 (PCE_avg_ = 2.74%) containing longer FCBS, as well as that of rigid fully conjugated PYT-C0 without FCBS (PCE_avg_ = 4.84%). We found that this significant difference in the PV performance is mainly due to enhanced exciton dissociation, suppressed monomolecular/trap-assisted recombination, optimized morphology, and high domain purity. Importantly, among the blends involving PBDB-T:PYT(S)-Cns, the blend with PYTS-C2 shows the best mechanical properties with a COS of 12.39% and a toughness of 2.09 MJ m^−3^, being at least two times higher than those of other blends with *P*_A_s of PYTS-C4, PYTS-C8, and PYT-C0 (COS = 6.03%, 4.77%, and 2.75%, and toughness = 0.68, 0.45, and 0.06 MJ m^−3^, respectively). This study demonstrates the success of integrating FCBS with the appropriate length into polymer conjugated backbone in improving solubility, mechanical ductility, and high efficiency of its corresponding all-PSCs.

## Experimental Section

### Materials

YBO-Br was synthesized according to the reference [47]. Thiophene, sulfur powder, 1,2-dibromoethane, 1,4-dibromobutane, 1,8-dibromooctane, *n*-butyl lithium, Pd_2_(dba)_3_, and P(*o*-Tol)_3_ were purchased from Sigma-Aldrich. 5,5'-bis(trimethylstannyl)-2,2'-bithiophene (T-C0-Sn) was purchased from Suzhou GR-chem Pharma Tech Co., Ltd. Poly[(2,6-(4,8-bis(5-(2-ethylhexyl)thiophen-2-yl)-benzo[1,2-*b*:4,5-*b*']dithiophene))-*alt*-(5,5-(1,3′-di-2-thienyl-5,7′-bis(2-ethylhexyl)benzo[1,2′-*c*:4′,5′-*c*′]dithiophene-4,8-dione))] (PBDB-T) was purchased from Brilliant Matters.

### Device Fabrication

The normal type all-PSCs (indium tin oxide (ITO)/poly(3,4-ethylenedioxy thiophene):polystyrene sulfonic acid (PEDOT:PSS, AI4083 from Heraeus)/active layer/interlayer/Ag) were fabricated by following processes. Poly[[2,7-bis(2-ethylhexyl)-1,2,3,6,7,8-hexahydro-1,3,6,8-tetraoxobenzo[lmn][3,8]phenanthroline-4,9-diyl]-2,5-thiophenediyl[9,9-bis[3'((N,N-dimethyl)-N-ethylammonium)]-propyl]-9H-fluorene-2,7-diyl]-2,5-thiophenediyl] (PNDIT-F3N-Br) was used as interfacial layer. ITO-coated glass substrates were washed by ultrasonication with deionized-water, acetone and isopropyl alcohol in series. The cleaned substrates were, dried for more than 1 h at 80 °C. A plasma treatment was proceeded for 10 min before spin-casting PEDOT:PSS solution. The PEDOT:PSS solution was spin-casted at 3000 rpm for 30 s, then thermally annealed at 165 °C for 15 min in ambient condition. Then, the samples were carried to a N_2_-filled glovebox. Next, the *P*_D_-*P*_A_ blend solutions with optimal concentration (12 mg mL^−1^) and *P*_D_:*P*_A_ ratio (1:1.2) were prepared in chloroform (CF). The solutions were stirred for at least 1 h at 55 °C before spin-coating. Then, the solutions were spin-casted onto the PEDOT:PSS-coated ITO substrates at 1750 rpm for 30 s, and the films were thermally annealed at 100 °C for 5 min. Then, the samples were stored in a high-vacuum chamber for 2 h to remove residual solvents in the films. Then, the PNDIT-F3N-Br solution (1 mg mL^−1^ in methanol) was spin-coated onto the active layer films with 3000 rpm for 30 s. Finally, Ag electrode (120 nm) was deposited by thermal evaporation in an evaporation chamber, under a high vacuum (~ 10^−6^ Torr) condition. The photoactive area of the all-PSC devices is 0.042 cm^2^, measured from the optical microscopy.

### Pseudo Free-standing Tensile Test

The films for the pseudo free-standing tensile test were prepared under the same condition with all-PSC fabrication. The films were spin-casted onto the PSS-coated glass substrate and cut into a dog-bone shape by a femtosecond laser. Then the films were floated onto the water surface, and attached to the grips by Van-der Walls interaction. The strain was applied with a fixed strain rate (0.8 × 10^−3^ s^−1^), and the tensile load values were measured by a load cell with high resolution (LTS-10GA, KYOWA, Japan). Elastic modulus was calculated using least square method for the slope of the linear region in stress-strain curve.

## Results and Discussion

### Synthesis and Characterization of PYT(S)-Cns

**Fig. 1:**
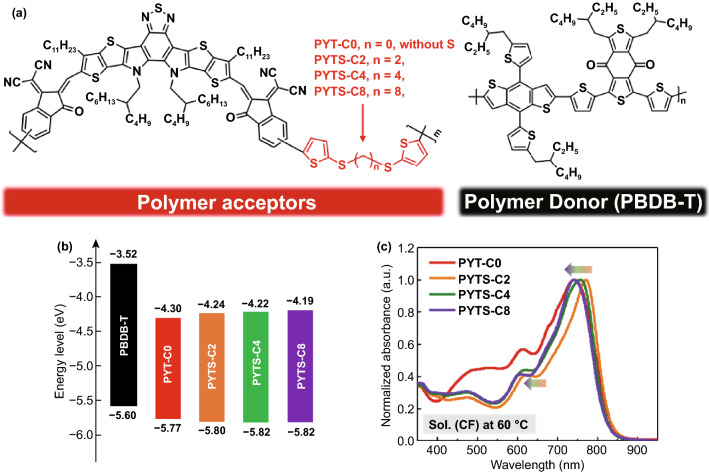
**a** Chemical structures of PYT(S)-Cn as the *P*_A_s and PBDB-T as the *P*_D_. **b** Molecular energy levels of PBDB-T and PYT(S)-Cns obtained from cyclic voltammetry measurements. **c** Normalized absorption spectra of the *P*_A_s in CF solution at 60 °C

The chemical structures of *P*_D_ and *P*_A_s used in this study are illustrated in Fig. 1a. PBDB-T was employed as *P*_D_ due to its matched energy levels and complementary optical absorption with those of a series of *P*_A_s [48]. We chose PYT-C0 as the reference *P*_A_ due to its fully conjugated rigid structure. Segments of thioalkyl with thiophene ends (TS-Cn) containing different lengths (highlighted in red in Fig. 1a) were incorporated into the backbone of PYT-C0 to provide different degrees of backbone flexibility between the conjugated YBO cores. The *P*_A_s were synthesized via Stille coupling polymerization of YBO-Br with T-C0-Sn or TS-Cn-Sn as FCBS units (Scheme S1). The resulting copolymer acceptors were named PYT-C0 and PYTS-Cn, where n = 2, 4, and 8, respectively, denoting the number of carbon atoms between the thiophene units in FCBSs. The number-average molecular weights (*M*_n_s) of PYTS-Cn polymers are in the range of 23 to 40 kg mol^−1^ as evaluated by gel permeation chromatography (GPC) (Table 1). In case of PYT-C0, the solubility was poorer compared to the FCBS-incorporated *P*_A_s, thus limiting its *M*_n_ growth during the polymerization. The decomposition temperatures (*T*_d_, 5% mass loss) of the *P*_A_s were found as high as 331–349 °C in thermogravimetric analysis (TGA) (Fig. S1), which indicates that all *P*_A_s have good thermal stability without negative effect by the incorporation of FCBS. The solubilities of the polymers were measured and summarized in Fig. S2 and Table S1. The electrochemical properties of the polymers were investigated via cyclic voltammetry (CV) measurements and outlined in Figs. 1b and S3**,** Table S2. The diagram of energy levels of *P*_A_s and *P*_D_ are illustrated in Fig. 1b, indicating that all *P*_A_s possess well-aligned HOMO and lowest unoccupied molecular orbital (LUMO) energy levels in connection with *P*_D_ for effective all-PSC operation. It was noted that PYT-C0 shows slightly higher HOMO and lower LUMO levels compared to PYTS-Cn with FCBSs, which is probably due to its slightly longer conjugated length without FCBS as indicated in density functional theory (DFT) simulation (see below) [45].

**Table 1:** Molecular weight-, optical-, and electrochemical-properties of the active materials used in this study

Polymer	*M*_n_ [kg mol ^–1^]	*PÐI*	*λ*_max_^sol, 20 °C^ [nm]^*a*^	*λ*_max_^sol, 60 °C^ [nm]	*λ*_max_^film^ [nm]^*a*^	*λ*_onset_^film^ [nm]^*a*^	*E*_g_^opt^ [eV]^*b*^
PBDB-T	43.0	2.78	610	-	572	673	1.84
PYT-C0	16.8	2.43	752	742	818	957	1.30
PYTS-C2	39.9	1.77	779	771	788	866	1.43
PYTS-C4	29.9	3.04	774	758	785	865	1.43
PYTS-C8	23.2	4.28	772	745	789	866	1.43

^a^ Obtained from Fig. S4; ^b^ calculated as 1240/*λ*_onset_^film^

The absorption spectra of the polymers were measured in both CF solutions and the solid states as shown in Fig. S4. The PBDB-T presents complementary absorption spectrum to those of the *P*_A_s. PYT-C0 exhibits noticeably blue-shifted absorption spectrum as compared to the other *P*_A_s with FCBS probably due to different molecular conformations and aggregation states in solutions. All *P*_A_s with FCBS showed similar *λ*_max_ and optical bandgap in films, which may be due to their comparably effective conjugation lengths and compact packing for all *P*_A_s in solid states (Fig. S4). However, for PYT-C0 film, its *λ*_max_ presents a considerable bathochromic shift of ca. 30 nm compared with other PYTS-Cn polymers, led by large aggregates originated from its rigid backbone when processed into films. Therefore, we assumed that the incorporation of bis(thioalkyl thiophene) could donate backbone flexibility and solution processability on *P*_A_s. To further investigate the effects of FCBS lengths on the aggregation properties of the *P*_A_s, temperature-dependent ultraviolet-visible absorption spectra of the *P*_A_s in CF solution were measured (Fig. S5) [34, 35, 49–51]. First, all *P*_A_s exhibited blue-shifted maximum absorption peaks and gradually decreased absorption intensity as temperature increased, which indicates that the *P*_A_s tended to aggregate at room temperature, and gradually disaggregated at higher temperatures [41, 52, 53]. In addition, such absolute blue-shifts for *P*_A_s from 20 to 60 °C were gradually enhanced with increasing of FCBS lengths in the order of 8 nm for PYTS-C2, 16 nm for PYTS-C4, and 27 nm for PYTS-C8. Correspondingly, a gradual blue-shift of maximum absorption peak for PYTS-Cns in CF solution at 60 ℃ was observed with increasing of FCBS length (Fig. 1c). This indicates that longer FCBSs enable molecular conformations and backbone-twisting of *P*_A_s to be more temperature-dependent. In addition, PYT-C0 in CF solution showed a less pronounced blue-shift of 10 nm from 20 to 60 °C, which could be due to its stronger intermolecular aggregation caused by rigid and planar conjugated skeleton. This factor makes it difficult to disaggregate at high temperature (Fig. S5). This is also supported by the fact that PYTS-C2, PYTS-C4, and PYTS-C8 exhibit much better solubilities in CF when the temperature increases from 20 to 50 °C, while the solubility of PYT-C0 remains poor even at 50 °C. As anticipated, the fully conjugated polymer PYT-C0 exhibited inferior solubility of 0.7 mg mL^−1^ in CF solution (at 50 °C) among *P*_A_s, which may be resulted from strong interchain aggregation of rigid backbone. For PYTS-Cns, the solubilities clearly increased when FCBSs incorporated in the polymer backbones, and they are varied depending on the lengths of FCBSs, measured as 39.8, 39.1, and 16.8 mg mL^−1^ for the PYTS-C2, PYTS-C4, and PYTS-C8 (Table S1), respectively. To investigate the effects of FCBS lengths on the solubilities of the *P*_A_s, grazing-incidence wide-angle X-ray scattering (GIWAXS) measurements were performed. PYTS-C8 showed larger crystal coherence lengths of *L*_c,100_^IP^ of 6.05 nm in the in-plane (IP) direction and *L*_c,010_^OOP^ of 2.14 nm in the out-of-plane (OOP) direction compared to those of PYTS-C2 (*L*_c,100_^IP^ = 0.88 nm, *L*_c,010_^OOP^ = 1.90 nm) and PYTS-C4 (*L*_c,100_^IP^ = 0.99 nm, *L*_c,010_^OOP^ = 1.80 nm). Moreover, PYT-C0 showed relatively large *L*_c,100_^IP^ of 5.04 nm and *L*_c,010_^OOP^ of 2.32 nm, which are shown in Fig. S6 and Table S3. These values indicate that the excessive aggregation and crystalline properties were effectively alleviated by the introduction of FCBS into PYT-C0, resulting in better solubilities for the PYTS-C2 and PYTS-C4 polymers. However, the presence of FCBSs longer than a certain length increased the aggregation and crystallinity again, leading to reduced solubility and increased crystal sizes, as proven in the case of PYTS-C8.

DFT calculations were performed to reveal the optimized molecular structures, frontier molecular orbitals, and origins of optical spectra differences for *P*_A_s with different lengths of FCBSs. The chemical model of two YBO cores linked one bithiophene or FCBS with methyl side chains instead of the bulky ones was chosen for simplifying the calculations. As illustrated in Figs. S7 and S8, respectively, PYT-C0 showed a relatively planar Z-shape configuration with a rigid backbone due to the centrosymmetric bithiophene linkage. This may be the reason for its strong intermolecular π-π aggregation and therefore relatively high crystallinity, resulted in its poor solubility (Tables S1 and S3). However, all PYTS-Cn with FCBS showed C-shape configurations and two YBO cores are found in different planes after structural optimizations, which might be the reasons for their entirely different aggregation behaviors from PYT-C0. Furthermore, the HOMO and LUMO of all PYTS-Cns exhibited similar charge distribution on only YBO cores instead of the FCBS linkage, which indicated that all PYTS-Cn have comparably effective conjugation lengths. As a comparison, HOMO of PYT-C0 is distributed along the whole YBO core and bithiophene linkage structure, which greatly promotes charge transfers (Fig. S8). It is expected that these different HOMO and LUMO distribution could induce the differences of absorption spectra between PYT-C0 and PYTS-Cns, as shown in theoretical calculation of their electronic excitations and absorption spectra (Figs. S4, S9 and S10, Table S4).

### Photovoltaic Properties

**Fig. 2:**
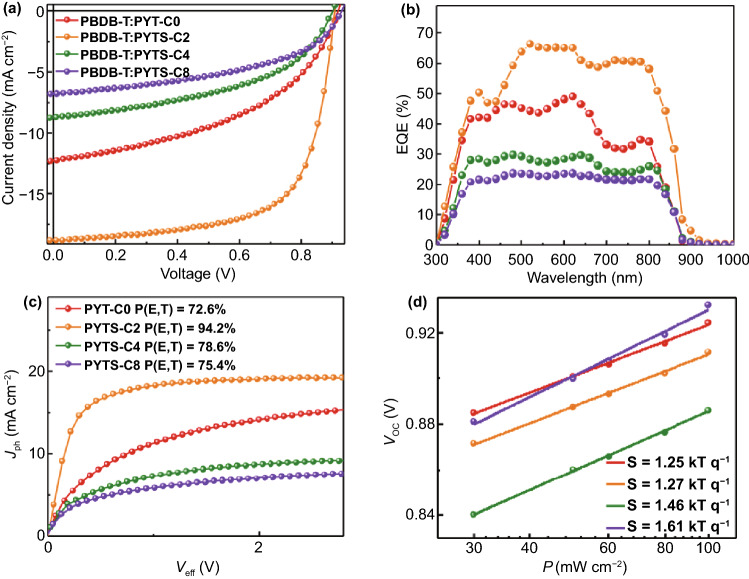
**a** *J*–*V* curves; **b** EQE response spectra; **c** *J*_ph_ vs. *V*_eff_ curves; **d** Dependences of *V*_oc_ on light intensities for the all-PSCs

Next, we investigated the PV properties of all-PSC devices with a normal geometry of ITO/PEDOT:PSS/active layer/PNDIT-F3N-Br/Ag. The detailed device structures and fabrication procedures are described in Supporting Information. The current density–voltage (*J*–*V*) curves under optimized condition and corresponding PV parameters are presented in Fig. 2a and Table 2. The PBDB-T:PYT-C0-based all-PSCs show a low PCE_avg_ of 4.84% with an open-circuit voltage (*V*_oc, avg_) of 0.92 V, and an average short-circuit current density (*J*_sc, avg_) of 11.99 mA cm^−2^. Notably, the all-PSCs based on the PBDB-T:PYTS-C2 blend exhibit a high PCE_avg_ of 11.20% (PCE_max_ = 11.37%) with its *J*_sc, avg_ of 18.45 mA cm^−2^, and a fill factor (FF_avg_) of 0.66. In contrast, the PCE_avg_s of PYTS-C4- and PYTS-C8-based all-PSCs with longer FCBS units decreased to 3.37% and 2.74%, respectively. These significant differences in PCEs indicate that the FCBS lengths in *P*_A_s have a significant impact on PV performance of all-PSCs. The external quantum efficiency (EQE) spectra are shown in Fig. 2b. The calculated *J*_sc_ values from the EQE spectra agreed well with the ones obtained from the *J-V* curves within 4% deviations (Table 2). Compared with those of other blends of PYT-C0, PYTS-C4, and PYTS-C8, PBDB-T:PYTS-C2 blends showed higher EQE responses over the full absorption range (300–900 nm). These results suggest that the active layer based on PYTS-C2 is optimal to obtain more efficient charge generations from both *P*_D_ and *P*_A_ absorption ranges than the others. Additionally, it was found that all-PSCs based on PYTS-C2 with higher molecular weight exhibits better PCE of 11.37% compared to that of PYTS-C2 with lower molecular weight (PCE = 9.11%) (Fig. S11 and Table S5), which may indicate that the high molecular weight polymer helps to promote morphology optimization and device performance improvement [53, 54].

**Table 2:** PV performances of the all-PSCs depending on the lengths of FCBSs in *P*_A_s

*P*_A_	*V*_oc_ [V]^*a*^	*J*_sc_ [mA cm^−2^]^*a*^	Calc. *J*_sc_ [mA cm^−2^]	FF^*a*^	PCE_avg (max)_ [%]^*a*^
PYT-C0	0.92 ± 0.01	11.99 ± 0.7	12.19	0.44 ± 0.02	4.84 ± 0.31 (5.15)
PYTS-C2	0.91 ± 0.01	18.72 ± 0.43	18.45	0.66 ± 0.02	11.20 ± 0.14 (11.37)
PYTS-C4	0.90 ± 0.01	8.01 ± 0.52	8.28	0.47 ± 0.01	3.37 ± 0.28 (3.73)
PYTS-C8	0.92 ± 0.01	6.33 ± 0.32	6.87	0.47 ± 0.00	2.74 ± 0.16 (2.97)

^a^ All parameters represent average values measured from five to ten all-PSC devices

In order to further clarify the reasons for the different PV performances of all-PSCs, we examined the charge generation, transport, and recombination mechanisms of the PBDB-T:*P*_A_ blends. First, the charge generation properties of blends were explored by calculating the exciton dissociation probabilities (*P*(E,T)) from the curves of photocurrent density (*J*_ph_) versus effective voltage (*V*_eff_) over the saturated photocurrent density (*J*_sat_, at *V*_eff_ = 2.5 V) (Fig. 2c). The *P*(E,T) values significantly increased from 72.6% for PBDB-T:PYT-C0 to 94.2% for PBDB-T:PYTS-C2 blend, and, then, decreased to 78.6% for PBDB-T:PYTS-C4 and 75.4% for PBDB-T:PYTS-C8. This suggests that the PBDB-T:PYTS-C2 blend has superior charge generation abilities at the donor:acceptor (D:A) interfaces to the other blend systems. Subsequently, the space charge limited current (SCLC) mobilities for the blend films were measured to investigate their charge transport properties (Table S6). Both hole mobility (*μ*_h_) and electron mobility (*μ*_e_) values of the PBDB-T:PYTS-C2 blend were higher than those of the other blends (*i.e.*, 1-order lower *μ*_e_ in magnitude), supporting its higher *J*_sc_ value.

The dependences of *V*_oc_ and *J*_sc_ on light intensity (*P*) were also measured to examine the charge recombination properties of the blends (Figs. 2d and S12). Generally, *V*_oc_ of all-PSC is proportional to the natural logarithm of *P* (*V*_oc_ = *S* × ln(*P*)), with the unit of *kT* *q*^−1^ (where *k* = Boltzmann constant, *T* = temperature and *q* = elementary charge), and its slope (*S*) approaches unity when no monomolecular or trap-assisted recombination occurs before charge collection on electrodes [55]. Notably, the *S* values for PYT-C0 and PYTS-C2 are similar (1.25 and 1.27 respectively), and gradually increase with the increasing lengths of FCBSs (1.46 for PYTS-C4, and 1.61 for PYTS-C8) (Fig. 2d). This indicates that incorporating short FCBS units in the *P*_A_s, particularly TS-C2, do not influence monomolecular or trap-assisted recombination of the all-PSCs, but incorporation of the longer FCBS units (TS-C4 and TS-C8) negatively affect the monomolecular or trap-assisted recombination properties. Additionally, *J*_sc_ and *P* follow the relationship of *J*_*sc*_ *∝* *P*^*α*^, where *α* as the power-law component would be close to 1 when the bimolecular recombination in devices is negligible [56]. The PBDB-T:PYT-C0 blend showed a lower *α* value of 0.88 than those of the other blends PYTS-Cns (*α* = 0.92–0.94). This indicates that the bimolecular recombination properties of the all-PSCs are improved through the incorporation of FCBSs in the *P*_A_s (Fig. S12). The above mentioned results from the charge generation and recombination properties support the increased *J*_sc_ and FF values in the PBDB-T:PYTS-C2 blends compared to the other PBDB-T:*P*_A_s blends.

### Thin-film Mechanical Properties

**Fig. 3:**
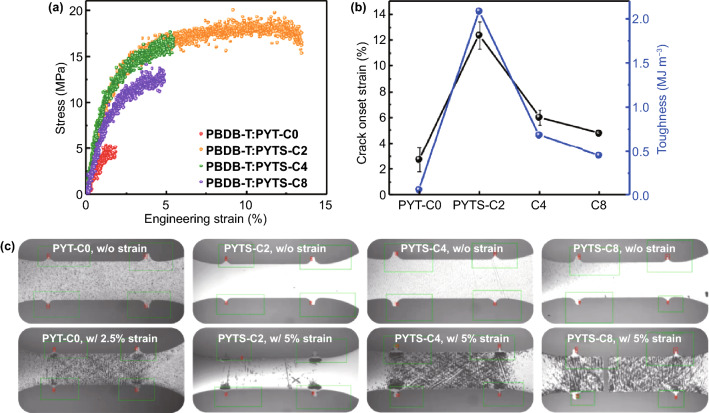
**a** *Stress–Strain* curves for the PBDB-T:*P*_A_s blends, and **b** Plots of COS and toughness values of the blends depending on the *P*_A_s. **c** Tensile-specimen images of the four blends during measurement without strain (upper images) and with 2.5% (PYT-C0) and 5% engineering strain (lower images). The green and red boxes indicate regimes for displacement tracking by digital image correction camera

The mechanical properties of the blend films were studied using a pseudo free-standing tensile test method [57, 58]. Thin-film samples of *P*_D_:*P*_A_s blends for mechanical testing were prepared under the same conditions as those of all-PSC blend films, and the tensile test results are shown in Fig. 3a-b and Table 3. Interestingly, PBDB-T:PYTS-C2 blend films showed superior COS of 12.39% with toughness of 2.09 MJ m^−3^, which are four times for the former and thirty times for the latter higher than those of PYT-C0 based blend films (COS = 2.75% and toughness = 0.06 MJ m^−3^). In addition, the COS and toughness values decreased rapidly with increased lengths of FCBSs (COS of 6.03% and 4.77%, toughness of 0.68 and 0.45 MJ m^−3^ for PBDB-T:PYTS-C4, and PBDB-T:PYTS-C8 blends, respectively). To further understand the difference in the mechanical properties of the blend films, the pristine films of polymer acceptors were also tested. However, except PYTS-C2, all other polymer acceptor films are very brittle, and no mechanical data can be obtained, which may relate to their relatively poor film-forming ability and strong crystallinity. The COS and toughness value for PYTS-C2 were 2.81% and 0.34 MJ m^−3^, respectively (Fig. S13 and Table S7). This indicates that the short FCBS length from TS-C2 incorporated into the conjugated polymer backbone could be optimal to afford simultaneously improved PV performance and mechanical properties.

**Table 3:** Mechanical properties of the PBDB-T:*P*_A_s blends measured from the pseudo free-standing tensile test

Blend	*E* [Mpa]^*a*^	COS [%]^*a*^	Toughness [MJ m^–3^]^*a*^
PBDB-T:PYT-C0	514	2.75	0.06
PBDB-T:PYTS-C2	649	12.39	2.09
PBDB-T:PYTS-C4	914	6.03	0.68
PBDB-T:PYTS-C8	626	4.77	0.45

^a^ All parameters represent average values from 3 samples

From the optical microscopy (OM) images of the tensile bars before testing, it can be seen clearly different initial morphologies of the blends depending on the *P*_A_s (Fig. 3c, upper images). The PBDB-T:PYT-C0 blend showed locally formed and large-sized defects in its film (grey dots), whereas the PBDB-T:PYTS-C2 blend exhibited more uniform and smoother surfaces with very few defects or agglomerates in the non-strain image. In comparison, the PBDB-T:PYTS-C4 and PBDB-T:PYTS-C8 blends displayed defects and agglomerates throughout the films, but with a smaller size than those of the PBDB-T:PYT-C0 blend. These different initial morphologies have influences to their crack propagation processes in the images of the strained films (Fig. 3c, lower images). The PBDB-T:PYT-C0, PBDB-T:PYTS-C4, and PBDB-T:PYTS-C8 blends showed many cracks being propagated around defect sites. These original cracks and defects in films accelerated the mechanical failures under further strains due to stress concentrations [33]. In contrast, the PBDB-T:PYTS-C2 blend exposed no such crack propagations, indicating that the uniform film is able to efficiently dissipate the mechanical stresses under the strains. The combined results of mechanical tests and OM images demonstrate that the incorporation of FCBS of appropriate length (*i.e.* two carbon atoms in this work) in the polymer acceptors not only provides the flexibility to polymer chains, but also allows the formation of optimal blend morphology without excessive aggregation and phase separation, bringing high mechanical strength and stretchability for the active layers.

### Morphological Properties

**Fig. 4:**
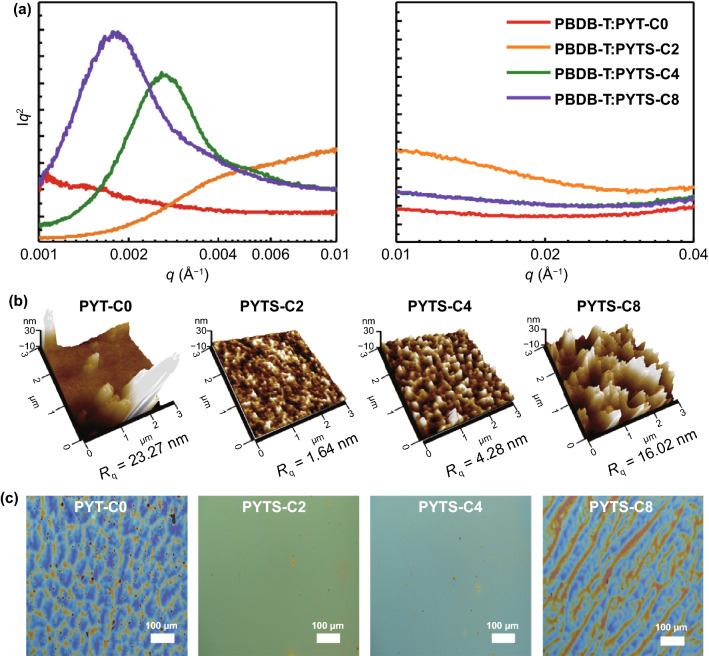
**a** RSoXS profiles, **b** AFM 3D height images, and **c** OM images of the PBDB-T:*P*_A_s blend films

To further explore the origins of the different PV- and mechanical-properties of the PBDB-T:*P*_A_ blends, morphology studies were examined with combined measurements of resonant soft X-ray scattering (RSoXS), atomic force microscopy (AFM), GIWAXS, and OM, as shown in Fig. 4, Table 4 and Fig. S14. First, the RSoXS profiles were obtained under a beam energy of 284.4 eV, which can maximize the material contrast between *P*_D_ and *P*_A_s [59, 60]. Relative domain purity, which is proportional to square root of integrated scattering intensity, was calculated for more quantitative analysis [59, 61]. All blends showed distinguishable peaks and significantly different domain-spacings and -purities (Fig. 4a and Table 4). As a result of increasing FCBS lengths in *P*_A_s, *d*-spacing values gradually increased, while the domain purities decreased. The PBDB-T:PYTS-C2 blend exhibited a small domain spacing (*d*-spacing = 61 nm) and a high relative domain purity of 1.00, compared with those of PBDB-T:PYTS-C4 blend (*d*-spacing = 242 nm, relative domain purity = 0.79) and PBDB-T:PYTS-C8 blend (*d*-spacing = 349 nm, relative domain purity = 0.82). It suggests that the presence of TS-C2 FCBS in *P*_A_ is effective to afford a smaller domain size and higher domain purity, thereby promoting efficient charge separation and charge transportation of all-PSCs [53, 54]. A similar trend of the surface roughness of PBDB-T:*P*_A_s blends depending on the lengths of FCBSs was observed in the AFM 3D height images (Fig. 4b and Table 4). The root-mean-square average roughness (*R*_q_) for PBDB-T:PYTS-C2, PBDB-T:PYTS-C4, and PBDB-T:PYTS-C8 blends were 1.6, 4.3, and 16.0 nm, respectively. This indicates that the further increase of FCBS lengths induces large phase segregation between *P*_D_ and *P*_A_s, which is also consistent with not only the decreased solubility in the case of PYTS-C8, but also their decreased charge generation and transport, therefore lower *J*_sc_ and FF, and finally lower PCEs as discussed earlier. On the other hand, the PYT-C0 based blend film did not show a distinct peak in RSoXS and exhibited an extremely large *R*_q_ of 23.3 nm in the AFM height image. We speculate that the peak of the PYT-C0 based blend film could not be detected in the measured *q* range of RSoXS due to its too large domain size. Furthermore, the OM images also showed a very non-uniform film with large aggregates in the PBDB-T:PYT-C0 blend (Fig. 4c). In contrast to the very rough and non-uniform surface of PBDB-T:PYTS-C8 blend film, the PBDB-T:PYTS-C2 and PBDB-T:PYTS-C4 blend films displayed a relatively uniform surface. Therefore, the results from the AFM, RSoXS and OM measurements show the same trend for the PBDB-T:*P*_A_ blend films.

**Table 4:** Morphological characteristics of the PBDB-T:*P*_A_s blends

Blend [w/ PBDB-T]	*d*-spacing [nm]^*a*^	Relative domain purity^*a*^	*R*_q_^AFM^ [nm]^*b*^
PYT-C0	–	–	23.3
PYTS-C2	61	1	1.6
PYTS-C4	242	0.79	4.3
PYTS-C8	349	0.82	16.0

^a^ Estimated from the RSoXS profiles; ^b^ Obtained from AFM height images

Another investigation of different blends morphology depending on the *P*_A_s was performed by comparing the crystalline properties of PBDB-T:*P*_A_s blends using the GIWAXS measurements (Fig. S14 and Table S8). It is found that the *L*_c_ values of the (100) peaks in the IP direction increased from 10.39 nm for PBDB-T:PYTS-C2 to 11.09 nm for PBDB-T:PYTS-C4 and 13.04 nm for PBDB-T:PYTS-C8. In comparison, the PBDB-T:PYT-C0 blend shows a relatively high IP *L*_c_ value of 11.32 nm. The *L*_c_ values for the (010) peaks in the OOP direction for all PBDB-T:*P*_A_ blends showed a similar trend with IP (100) peaks (Table S8). These results indicate that incorporation of short TS-C2 FCBS could effectively decrease the crystallinity of the fully conjugated *P*_A_ in the blends. However, longer FCBSs than TS-C2 in *P*_A_s resulted in rather increased crystallinity in the blends. Additionally, the interfacial tensions between *P*_A_s and *P*_D_ were calculated by measuring the pristine film contact angle of materials using glycerol and water droplets by means of Wu method (Table S9). PYTS-C2 and PYTS-C4 films showed lower interfacial tension values of 1.38 and 1.44 mN m^−1^ when paired with PBDB-T than those of the PYT-C0-based (6.31  mN m^−1^) and PYTS-C8-based blends (2.82 mN m^−1^). This supports that PYTS-C2 and PYTS-C4 have better compatibility with PBDB-T compared to the remaining ones [62]. These lower interfacial tensions of PYTS-C2 and PYTS-C4 could reduce the degree of their phase-separations and decrease the domain size in the blend [63].

**Fig. 5:**
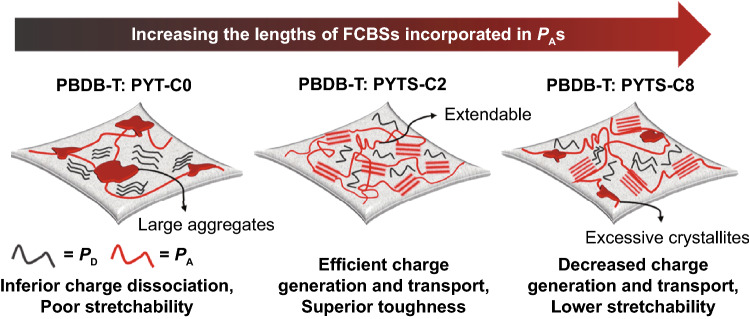
Schematic illustration of the PBDB-T:PYT(S)-Cn blend morphologies with different length of FCBS

Based on the evaluated results of the above morphology analyses, PBDB-T:PYT-C0 blend shows large aggregates and segregated domains compared with those of PBDB-T:PYTS-Cn blends. These are mainly due to the low solubility of PYT-C0 polymers inducing the excessively large aggregates and poor mixing with *P*_D_. In this case, the excessively segregated blend morphology decreases charge dissociation, increases bimolecular recombination and results in very poor mechanical stretchability in the blend (the left illustration of Fig. 5). On the other hand, when too long FCBS (over C4) is incorporated into the *P*_A_s, the blend film also exhibits severe phase-separation between *P*_D_ and *P*_A_ due to their less compatibility with *P*_D_, even though the *P*_A_s showed reduced aggregate properties. These separated domains with low compatibilities can hinder the charge transfer to other domains, limiting the PV performances of all-PSCs. In addition, the sharp and weak interfaces between the domains could provide crack propagation pathways to degrade mechanical properties (the right illustration of Fig. 5).

Employing the optimal length of FCBS in *P*_A_ affords the excellent solubility in case of PYTS-C2 to prevent the formation of excessive aggregates and induce the superior compatibility with *P*_D_. This morphology enables efficient charge separation and transport. The small domain sizes of the PBDB-T:PYTS-C2 film greatly reduce the crack propagation route and promote the extension of films without significant cracking during the stretching (the middle illustration of Fig. 5). Therefore, the PBDB-T:PYTS-C2 based all-PSCs showed not only high PCE of 11.37% but also superior mechanical properties (COS of 12.39%, toughness of 2.09 MJ m^−3^). Overall, the length of FCBS integrated in *P*_A_ is an important factor in (i) controlling the *P*_A_ solubility; and (ii) increasing the compatibility between *P*_D_ and *P*_A_, to achieve high PV performance and superior stretchability in all-PSCs.

## Conclusions

In summary, we developed a new series of *P*_A_s by incorporating FCBS units with different lengths into the rigid backbone, thereby simultaneously enhancing the PV performance and mechanical robustness of all-PSCs. It was found that the incorporation of certainly short FCBS (PYTS-C2) endowed increased backbone flexibility, and thus prevented excessive aggregation, leading to the significantly increased solubility to the resulting *P*_A_s. However, the longer FCBSs (PYTS-C4 and PYTS-C8) increased intermolecular and/or intramolecular π-π stacking, causing decreased solubility and excessive aggregation, which resulted in poor compatibility with donor polymers and suboptimal morphology in forming films. As a result, the all-PSC based on PBDB-T:PYTS-C2 exhibited a PCE of 11.37%, which is superior to those of the devices based on PYT-C0 without FCBS and *P*_A_s with longer FCBSs. Moreover, the improved morphology of PBDB-T:PYTS-C2 blend realized higher mechanical stretchability and robustness with a COS of 12.39% and toughness of 2.09 MJ m^–3^, compared with PBDB-T:PYT-C0 and PBDB-T:PYTS-Cn blends. Our findings demonstrate that introducing appropriate FCBS with short length, particularly TS-C2 for PYTS-C2, can simultaneously enhance the photovoltaic performance and mechanical robustness of the devices. Overall, this work inspires molecular design with rational FCBS selections for realizing highly efficient and mechanically robust all-PSCs that are appropriate for stretchable and flexible electronics.

## Supplementary Information

Below is the link to the electronic supplementary material.

Supplementary file1 (DOCX 10594 KB)
