# Supplementary material for: Effects of Flexible Conjugation-Break Spacers of Non-Conjugated Polymer Acceptors on Photovoltaic and Mechanical Properties of All-Polymer Solar Cells
Source: Nanomicro Lett. 2022 Aug 13;14:164. doi: 10.1007/s40820-022-00884-8 (PMC9375791; doi:10.1007/s40820-022-00884-8)
Supplement: Supplementary file 1 — Supplementary file1 (DOCX 10594 KB) [file 40820_2022_884_MOESM1_ESM.docx]

**Supporting information for**

**Effects of Flexible Conjugation-Break Spacers of Non-Conjugated Polymer Acceptors on Photovoltaic and Mechanical Properties of All-Polymer Solar Cells**

Qiaonan Chen^1, 2^, Yung Hee Han^3^, Leandro R. Franco^4^, Cleber F. N. Marchiori^4^, Zewdneh Genene^2^, C. Moyses Araujo^4, 7^, Jin-Woo Lee^3^, Tan Ngoc-Lan Phan^3^, Jingnan Wu^2,5^, Donghong Yu^5, 8^, Dong Jun Kim^6^, Taek-Soo Kim^6^, Lintao Hou^1,^ *, Bumjoon J. Kim^3,^ *, and Ergang Wang^2, 9,^ *

^1^Department of Physics, Siyuan Laboratory, Guangzhou Key Laboratory of Vacuum Coating Technologies and New Energy Materials, Jinan University, Guangzhou 510632, China

^2^Department of Chemistry and Chemical Engineering, Chalmers University of Technology, Göteborg, SE-412 96, Sweden

^3^Department of Chemical and Biomolecular Engineering, Korea Advanced Institute of Science and Technology (KAIST), Daejeon 34141, Republic of Korea

^4^Department of Engineering and Physics, Karlstad University, Karlstad 65188, Sweden

^5^Department of Chemistry and Bioscience, Aalborg University, Aalborg DK-9220, Denmark

^6^Department of Mechanical Engineering, Korea Advanced Institute of Science and Technology (KAIST), Daejeon 34141, Republic of Korea

^7^Materials Theory Division, Department of Physics and Astronomy, Uppsala University, Uppsala 75120, Sweden

^8^Sino-Danish Center for Education and Research, Aarhus, DK-8000, Denmark

^9^School of Materials Science and Engineering, Zhengzhou University, Zhengzhou 450001, China

Qiaonan Chen and Yung Hee Han contributed equally to this work.

*Corresponding author. E-mail: Lintao Hou, thlt@jnu.edu.cn; Bumjoon J. Kim, bumjoonkim@kaist.ac.kr; Ergang Wang, ergang@chalmers.se

**Table of Contents**

**Experimental Section**

**Synthesis of the acceptor polymers**

**Supporting Figures**

**Fig. S1** TGA of PYT(S)-Cn (n=0, (without C-S bonds) and 2, 4, and 8 carbon atoms).

**Fig. S2** Solubilities of the aggregated polymers (with particles of less than 200 nm in diameter) in CF solutions at 50 ℃.

**Fig. S3** Cyclic voltammograms of *P*_A_s.

**Fig. S4** Normalized absorption spectra of *P*_A_s in (**a**) CF solutions at room temperature, and (**b**) in pristine films.

**Fig. S5** Temperature-dependent UV-Vis absorption spectra for *P*_A_s in CF at a concentration of 0.02 mg mL^-1^.

**Fig. S6** GIWAXS linecut profiles of the pristine polymers in (**a**) IP and (**b**) OOP directions.

**Fig. S7** Vacuum optimized geometries of PYT(S)-Cn (n=0, (without C-S bonds) and 2, 4, and 8 carbon atoms) using TPSSh/6-31G(d).

**Fig. S8** HOMO and LUMO orbitals of PYT(S)-Cn (n=0, (without C-S bonds) and 2, 4, and 8 carbon atoms) using TPSS/6-311G(d,p).

**Fig. S9** Electronic excitations and absorption spectra of PYT(S)-Cn (n=0 (no C-S bonds either), 2, 4, and 8 carbon atoms) in CF solution.

**Fig. S10** Comparison of absorption maximum for PYT(S)-Cn (n=0 (no C-S bonds either), 2, 4, and 8 carbon atoms) in CF at 20 ℃ (blue line), in film (red line) and by the Lorentzian convolution of the TD-TPSSh/6-311G(d,p) excitations (yellow line).

**Fig. S11** *J–V* curves of all-PSCs based on PBDB-T:PYTS-C2 with different molecular weight and PDI of PYTS-C2.

**Fig. S12** Dependences of *J*_sc_ on light intensities of the all-PSCs.

**Fig. S13** *Stress–Strain* curves for the pristine PYTS-C2 film.

**Fig. S14** GIWAXS linecut profiles of the PBDB-T:*P*_A_s blends in (**a**) IP and (**b**) OOP directions.

**Fig. S15-26** NMR Spectra of the TS-Cn and TS-Cn-Sn.

**Supporting Tables**

**Table S1** Solubilities of polymers

**Table S2** Electrochemical parameters of *P*_A_s from cyclic voltammograms

**Table S3** GIWAXS characteristics of the pristine polymers

**Table S4** The first 5 electronic excitations of PYT(S)-Cn (n=0 (no C-S bonds either), 2, 4, and 8 carbon atoms) calculated with TD-DFT and the absorption maximum obtained from measurements

**Table S5** PV performances of the all-PSCs depending on the molecular weight of PYTS-C2

**Table S6** SCLC mobilities of the PBDB-T:*P*_A_s blends

**Table S7** Mechanical properties of the PYTS-C2 film measured from the pseudo free-standing tensile test

**Table S8** GIWAXS characteristics of the PBDB-T:*P*_A_s blends

**Table S9** Contact angle data of PBDB-T and *P*_A_s

**Experimental Section**

**Characterization**

^1^H and ^13^C NMR spectra were recorded on Bruker AV 400 MHz FT-NMR spectrometer by using in CDCl_3_. Gel permeation chromatography (GPC) was carried out on an Agilent PL-GPC 220 integrated high-temperature GPC/SEC system equipped with refractive index and viscometer detectors and three sequential PLgel 10 μm MIXED-B LS 300 mm × 7.5 mm columns. The eluent was 1,2,4-trichlorobenzene and the operating temperature was 150 °C. The number-average molecular weights (*M_n_*s) were calculated relative to polystyrene standards (via calibration). The ultraviolet-visible (UV−Vis) spectra of the polymers were recorded on a UV-Vis-NIR Spectrophotometer of Agilent Technologies Cary Series. Thermogravimetric analysis (TGA) was conducted by a Mettler Toledo TGA/DSC 3+ STAR System instrument under nitrogen atmosphere at a heating rate of 10 ℃ min^-1^. Grazing incident wide angle X-ray scatter (GIWAXS) measurement was performed at Pohang Accelerator Laboratory (beamline = 9A) in Republic of Korea. The incidence angles for the GIWAXS were set to 0.12 - 0.14° for complete penetration of thin films. *L*_c_ values of the polymer films were calculated by means of Scherrer equation.

$$L_{c}= \frac{2\pi K}{\Delta_{q}}$$

(*K* = shape factor (0.9), Δ*_q_* = full width half maximum (FWHM) of scattered peaks, respectively.) A McScience K201 LAB55 solar simulator and Keithley 2400 SMU were used to determine the photovoltaic performance and properties of the OSCs under AM1.5G 100 mW cm^-2^. A McScience K3100 was used to calculate external quantum efficiency (EQE) spectra of the all-PSCs. A McScience K801SK302 standard cell was used to calibrate solar intensity.

**Solubility test**

We followed a method for the solubility test in the literature [1]. The solubilities of the pristine polymers were estimated in the chloroform (CF) solutions at 50 ℃. The polymers were dissolved into CF with a concentration of 40 mg mL^–1^ and heated to 60 ℃ to produce non-saturated solutions. Then, the temperature of the solutions was decreased to 50 ℃ for gaining over-saturated solutions and filtered through a polyvinylidene fluoride (PVDF) membrane having pores with a 200 nm diameter in order to obtain their exactly saturated solutions, which were evaporated in a vacuum oven for a day, and the mass of the remained polymers were measured to estimate the solubility of corresponding polymers at 50 ℃.

**Cyclic Voltammetry (CV)**

The CV of the *P*_D_ and *P*_A_s was recorded on a CH-Instruments 650A Electrochemical Workstation in a three-electrode configuration using Pt wires as both working- and counter-electrode, and a freshly activated Ag wire as Ag/Ag^+^ pseudo-reference electrode calibrated versus the Fc/Fc^+^ redox couple at the end of the measurements. A tetrabutylammonium hexafluorophosphate (Bu_4_NPF_6_) solution (0.1 M solution in anhydrous acetonitrile) was used as a supporting electrolyte, which was bubbled with N_2_ gas prior to each measurement to remove any dissolved oxygen. Polymer films were deposited onto the working electrode by drop-casting from chloroform solutions of 10 mg mL^-1^ polymers. The energy levels were calculated according to the formula LUMO = −(*E*_onset_^red^ + 5.13) eV and HOMO = −( *E*_onset_^ox^ + 5.13) eV, where the *E*_onset_^red^ and *E*_onset_^ox^ were determined from the reduction and oxidation onsets, respectively. HOMO and LUMO levels were estimated from the onset potentials by setting the oxidative onset potential of Fc/Fc^+^ vs. the normal hydrogen electrode (NHE) to 0.63 V [2], and the NHE vs. the vacuum level to 4.5 V [3].

**Space Charge Limited Current (SCLC) measurement**

The electron- and hole-only devices for the SCLC measurement have device architectures of ITO/ZnO/active layer/LiF/Al and ITO/PEDOT:PSS/active layer/Au, respectively. The pristine and blend films were spin-casted in the N_2_-filled glovebox, and the blend films were prepared in the same condition as that for all-PSC fabrication. Mott-Gurney equation was used to fit the *J*-*V* characteristics:

$J_{SCLC}\text{ }=\frac{9}{8}\varepsilon_{0}\varepsilon_{r}\mu(V^{2}/L^{3})$.

($\varepsilon_{0}$ = the free-space permittivity, $\varepsilon_{r}$ = the dielectric constant of the semiconductor, $\mu$ = the mobility, *V* = the applied voltage, and *L* = the thickness of the active layer.)

**Density Functional Theory (DFT) calculation**

DFT modelling of *P*_A_s (PYT-C0, PYTS-C2, PYTS-C4 and PYTS-C8) with methyl instead of long alkyl side chains on YBO core in chloroform was performed at the TPSSh [4]/(6-31G(d) and 6-311G(d,p)) [4, 5] theory level with the inclusion of solvent effects under the universal Solvation Model based on solute Density (SMD) [6], as implemented in Gaussian 16 (Rev. C.01) [7].

**Synthesis of the acceptor polymers**

**Scheme S1** Synthetic routes of the acceptor polymers of PYT(S)-Cn (n=0, (without C-S bonds) and 2, 4, and 8 carbon atoms).

**Synthesis of TS-Cn**

**TS-Cn** was synthesized according to the reference [8]. In a 250 mL dry flask, thiophene (**T**, 0.10 mol) and 150 mL dry tetrahydrofuran (THF) were mixed under nitrogen protection, and then the solution was bubbled with nitrogen for 10 min. The solution was cooled to -78 ℃, followed by addition of 40 mL of *n*-butyl lithium (0.10 mol, 2.5 M in hexane) dropwise. The reaction mixture was stirred at -78 ℃ for 1 h and then reacted at room temperature (RT) for another 1 h. Subsequently, sulfur powder (3.20 g, 0.10 mol) was added to the mixture at 0 ℃ and then react for 2 h at RT. Then, the 1,2-dibromoethane (or 1,4-dibromobutane or 1,8-dibromooctane) (0.04 mol) was added into the mixture at 0 ℃, and stirred at RT overnight. After that, the mixture was added with brine and extracted with diethyl ether twice. The organic layer was washed with brine for three times and dried with anhydrous Na_2_SO_4_. The solvent was removed under reduced pressure, and the crude product was purified by column chromatography with silica gel (300-400 mesh) using dichloromethane (DCM)/petroleum ether (PE) = 0.15: 1 as an eluent to afford the product **TS-Cn** as colourless oils.

**TS-C2** (5.97 g, yield 58%) ^1^H NMR (400 MHz, CDCl_3_, TMS), δ (ppm): 7.35 (dd, *J* = 5.4, 1.3 Hz, 2H), 7.11 (dd, *J* = 3.5, 1.3 Hz, 2H), 6.97 (dd, *J* = 5.4, 3.5 Hz, 2H), 2.95 (s, 4H). ^13^C NMR (100 MHz, CDCl_3_, TMS), δ (ppm): 134.5, 134.4,133.1, 130.0, 129.9, 127.8, 38.0.

**TS-C4** (9.17 g, yield 64%). ^1^H NMR (400 MHz, CDCl_3_, TMS), δ (ppm): 7.33 (dd, *J* = 5.3, 1.3 Hz, 2H), 7.10 (dd, *J* = 3.5, 1.3 Hz, 2H), 6.97 (dd, *J* = 5.3, 3.5 Hz, 2H), 2.87-2.71 (m, 4H), 1.79-1.65 (m, 4H). ^13^C NMR (100 MHz, CDCl_3_, TMS), δ (ppm): 134.5, 133.7, 129.3, 127.6, 38.5, 28.1.

**TS-C8** (9.48 g, yield 69%).^1^H NMR (400 MHz, CDCl_3_, TMS), δ (ppm): 7.33 (dd, *J* = 5.4, 1.3 Hz, 2H), 7.10 (dd, *J* = 3.5, 1.3 Hz, 2H), 6.97 (dd, *J* = 5.3, 3.5 Hz, 2H), 2.87-2.74 (m, 4H), 1.66-1.56 (m, 4H), 1.29-1.21 (m, 4H). ^13^C NMR (100 MHz, CDCl_3_, TMS), δ (ppm): 135.0, 133.4, 129.0, 127.6, 39.0, 29.4, 29.1, 28.4.

**Synthesis of TS-C2-Sn**

**TS-Cn-Sn** was synthesized according to the reference [8]. In a 250 mL dry flask, **TS-C2** (2.44 g, 10 mmol) and 60 mL dry THF were mixed under nitrogen protection, and then the solution was bubbled with nitrogen for 10 min. The solution was cooled to -78 ℃, and then 10 mL of *n*-butyl lithium (25 mmol, 2.5 M in hexane) was added dropwise. The reaction mixture was stirred at -78 ℃ for 1 h, producing a suspension with white solids and then reacted at room RT for another 1 h, becoming a suspension with light pink solids. Subsequently, the reaction was cooled to -78 ℃ and 30 mL of chlorotrimethylstannane (30 mmol, 1.0 M in THF) was added into the mixture, resulting in a yellow solution. Then, the reaction mixture was stirred overnight at RT. After that, the mixture was added with water and extracted with diethyl ether twice. The organic layer was washed with water for five times and dried with anhydrous Na_2_SO_4_. The solvent was removed under reduced pressure to produce the crude product as a yellow oil. Afterwards, the crude product was put in the fridge and became solids, which was further washed with cold ethanol for five times and finally produced compound **TS-C2-Sn** (3.33 g, yield 57%) as white solids. ^1^H NMR (400 MHz, CDCl_3_, TMS), δ (ppm): 7.18 (d, *J* = 3.2 Hz, 2H), 7.05 (d, *J* = 3.3 Hz, 2H), 2.97 (s, 4H), 0.37 (s, 9H). ^13^C NMR (100 MHz, CDCl_3_, TMS), δ (ppm): 143.8, 138.3,135.7, 134.9, 134.8, 38.0, -8.1.

**Synthesis of TS-C4-Sn**

In a 250 mL dry flask, **TS-C4** (2.86 g, 10 mmol) and 60 mL dry THF were mixed under nitrogen protection, and then the solution was bubbled with nitrogen for 10 min. The solution was cooled to -78 ℃, and then 10 mL of *n*-butyl lithium (25 mmol, 2.5 M in hexane) was added dropwise. The reaction mixture was stirred at -78 ℃ for 1 h, producing a suspension with orange solids and then reacted at RT for another 1 h, becoming pink solution. Subsequently, the reaction was cooled to -78 ℃ and 30 mL of chlorotrimethylstannane (30 mmol, 1.0 M in THF) was added into the mixture, resulting a light orange solution. Then, the reaction mixture was stirred overnight at RT. Afterwards, the mixture was added with water and extracted with diethyl ether twice. The organic layer was washed with water for five times and dried with anhydrous Na_2_SO_4_. The solvent was removed under reduced pressure to produce the crude product as an oil. After that, the crude product was put in the fridge and became solids, which was further washed with cold ethanol for five times and finally produced compound **TS-C4-Sn** (4.26 g, yield 70%) as light pink solids. ^1^H NMR (400 MHz, CDCl_3_, TMS), δ (ppm): 7.17 (d, *J* = 3.3 Hz, 2H), 7.05 (d, *J* = 3.3 Hz, 2H), 2.84-2.75 (m, 4H), 1.80-1.68 (m, 4H), 0.36 (s, 9H). ^13^C NMR (100 MHz, CDCl_3_, TMS), δ (ppm): 142.7, 140.2, 135.6, 133.8, 38.9, 29.4, 28.1, -8.1.

**Synthesis of TS-C8-Sn**

In a 250 mL dry flask, **TS-C8** (3.42 g, 10 mmol) and 60 mL dry THF were mixed under nitrogen protection, and then the solution was bubbled with nitrogen for 10 min. The solution was cooled to -78 ℃, and then 10 mL of *n*-butyl lithium (25 mmol, 2.5 M in hexane) was added dropwise. The reaction mixture was stirred at -78 ℃ for 1 h, producing a suspension with solids and then reacted at RT for another 1 h, becoming pink solution. Subsequently, the reaction was cooled to -78 ℃ and 30 mL of chlorotrimethylstannane (30 mmol, 1.0 M in THF) was added into the mixture, resulting in a light orange solution. Then, the reaction mixture was stirred overnight at RT. After that, the mixture was added with water and extracted with diethyl ether twice. The organic layer was washed with water for five times and dried with anhydrous Na_2_SO_4_. The solvent was removed under reduced pressure to produce the crude product as an oil. After that, the crude product was put in the fridge and became solids, which was further washed with cold ethanol for five times and finally produced compound **TS-C8-Sn** (3.12 g, yield 45%) as light pink solids. ^1^H NMR (400 MHz, CDCl_3_, TMS), δ (ppm): 7.17 (d, *J* = 3.3 Hz, 2H), 7.06 (d, *J* = 3.3 Hz, 2H), 2.84-2.74 (m, 4H), 1.67-1.57 (m, 2H), 1.46-1.33 (m, 2H), 1.33-1.24 (m, 2H), 0.36 (s, 9H). ^13^C NMR (100 MHz, CDCl_3_, TMS), δ (ppm): 142.6, 140.3,135.6, 133.7, 39.0, 29.6, 29.1, 28.5, -8.1.

**Synthesis of polymer PYT-C0**

**YBO-Br** (200.0 mg, 0.121 mmol), **T-C0-Sn** (59.6 mg, 0.121 mmol), Pd_2_(dba)_3_ (2.2 mg, 0.0024 mmol) and P(*o*-Tol)_3_ (3.0 mg, 0.0097 mmol) were added to a 12 mL dry vial. After three cycles of alternative vacuum and nitrogen treatment for ideal removal of oxygen, 10 mL dry toluene was added into the vial. The solution was stirred vigorously at 100 ℃ for 22 h. The mixture was precipitated in methanol and filtrated. The polymer was dissolved in hot *o*-dichlorobenzene (*o*-DCB) and the solution was filtered through a short silica gel (70-230 mesh) column under pressure to remove the low molecular weight polymer and metal catalyst. The collected *o*-DCB solution was precipitated into methanol followed by filtration and drying in vacuum overnight yielded a dark solid (130.0 mg, yield 65%). *M_n_* = 16.8 kDa, *M_w_* = 40.8 kDa, PDI = 2.43.

**Synthesis of PYTS-C2**

**YBO-Br** (200.0 mg, 0.121 mmol), **TS-C2-Sn** (71.8 mg, 0.121 mmol), Pd_2_(dba)_3_ (2.2 mg, 0.0024 mmol) and P(*o*-Tol)_3_ (3.0 mg, 0.0097 mmol) were added to a 12 mL dry vial. After three cycles of alternative vacuum and nitrogen treatment for ideal removal of oxygen, 10 mL dry toluene was added into the vial. The solution was stirred vigorously at 100 ℃ for 22 h. There were no sediments directly obtained as products from solution. The mixture was then precipitated in methanol and filtrated. The solids were dissolved in hot CF and the solution was filtered through a short silica gel (70-230 mesh) column under pressure to remove the low molecular weight polymer and metal catalyst. The collected CF solution was precipitated into methanol, followed by filtration, and drying in a vacuum-oven overnight yielded a dark solid (118.0 mg, yield 63%). *M_n_* = 39.9 kDa, *M_w_* = 70.6 kDa, PDI = 1.77.

**Synthesis of polymer PYTS-C4**

**YBO-Br** (200.0 mg, 0.121 mmol), **TS-C4-Sn** (75.3 mg, 0.121 mmol), Pd_2_(dba)_3_ (2.2 mg, 0.0024 mmol) and P(*o*-Tol)_3_ (3.0 mg, 0.0097 mmol) were added to a 12 mL dry vial. After three cycles of alternative vacuum and nitrogen treatment for ideal removal of oxygen, 10 mL dry toluene was added into the vial. The solution was stirred vigorously at 100 ℃ for 22 h. The main products precipitated on the wall of the vial. The solids were dissolved in hot *o*-DCB and the solution was filtered through a short silica gel (70-230 mesh) column under pressure to remove the low molecular weight polymer and metal catalyst. The collected *o*-DCB solution was precipitated into methanol, followed by filtration, and drying in a vacuum oven overnight yielded a dark solid (45.0 mg, yield 21%). *M_n_* = 29.9 kDa, *M_w_* = 91.0 kDa, PDI = 3.04.

**Synthesis of polymer PYTS-C8**

**YBO-Br** (200.0 mg, 0.121 mmol), **TS-C8-Sn** (91.3 mg, 0.121 mmol), Pd_2_(dba)_3_ (2.2 mg, 0.0024 mmol) and P(*o*-Tol)_3_ (3.0 mg, 0.0097 mmol) were added to a 12 mL dry vial. After three cycles of alternative vacuum-and-nitrogen treatment for ideal removal of oxygen, 10 mL dry toluene was added into the vial. The solution was stirred vigorously at 100 ℃ for 22 h. The main products precipitated on the wall of the vial. The products in solution were found to be low molecular-weight fraction of the polymers (*M_n_* = 2.9 kDa), which wasn’t used for further study. The solids were dissolved in hot *o*-DCB and the solution was filtered through a short silica gel (70-230 mesh) column under pressure to remove the metal catalyst. The collected *o*-DCB solution was precipitated into methanol, followed by filtration, and drying in vacuum overnight yielded a dark solid (91.0 mg, yield 41%). *M_n_* = 23.2 kDa, *M_w_* = 99.4 kDa, PDI = 4.28.

**Supporting Figures & Tables**


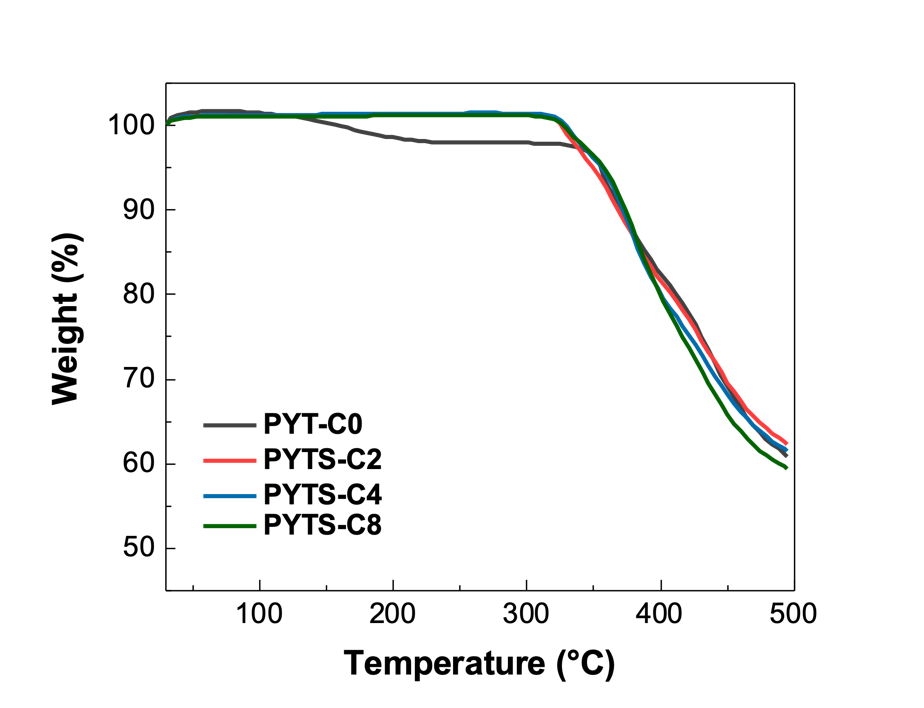


**Fig. S1** TGA of PYT(S)-Cn (n=0, (without C-S bonds) and 2, 4, and 8 carbon atoms).


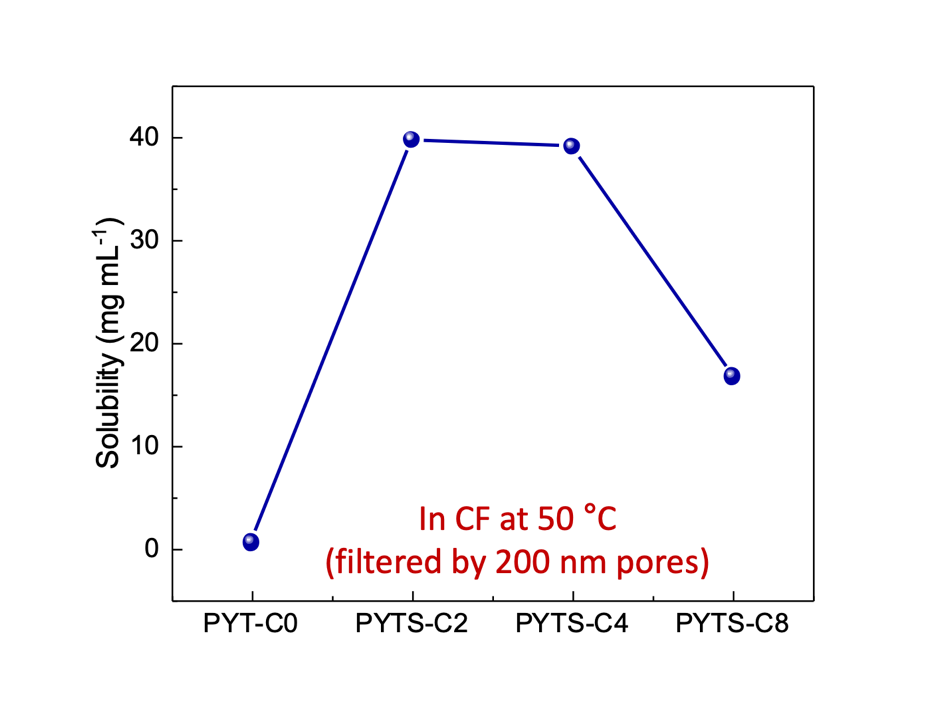


**Fig. S2** Solubilities of the aggregated polymers (with particles of less than 200 nm in diameter in CF solutions at 50 ℃.

**Table S1**. Solubilities of polymers

| **Polymer** | **Solubility**  **[mg mL^–1^]** *^a^* |
| --- | --- |
| PYT-C0 | 0.7 |
| PYTS-C2 | 39.8 |
| PYTS-C4 | 39.1 |
| PYTS-C8 | 16.8 |

*^a^* in CF solutions at 50 ℃ (filtered by pores with a 200 nm diameter).

**
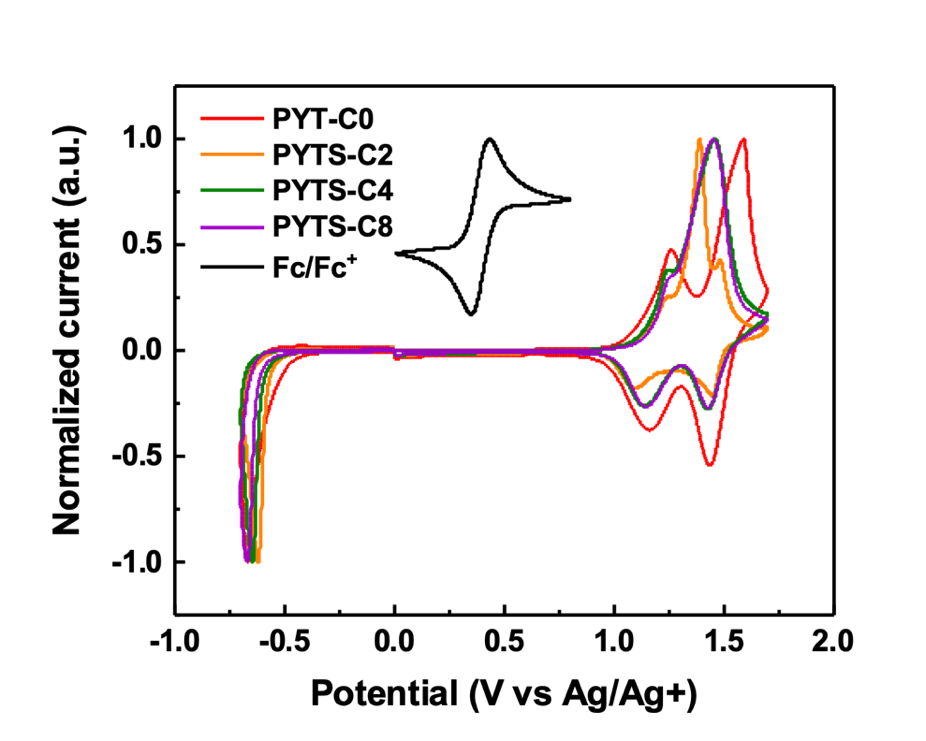
**

**Fig. S3** Cyclic voltammograms of *P*_A_s.

**Table S2** Electrochemical parameters of *P*_A_s from cyclic voltammograms

| **Polymer** | **CV** | | | |
| --- | --- | --- | --- | --- |
|  | **E_onset,Red_**  **[V]** | **LUMO**  **[eV]** | **E_onset,Ox_**  **[V]** | **HOMO**  **[eV]** |
| PYT-C0 | -0.83 | -4.30 | 0.64 | -5.77 |
| PYTS-C2 | -0.89 | -4.24 | 0.67 | -5.80 |
| PYTS-C4 | -0.91 | -4.22 | 0.69 | -5.82 |
| PYTS-C8 | -0.94 | -4.19 | 0.72 | -5.82 |

**
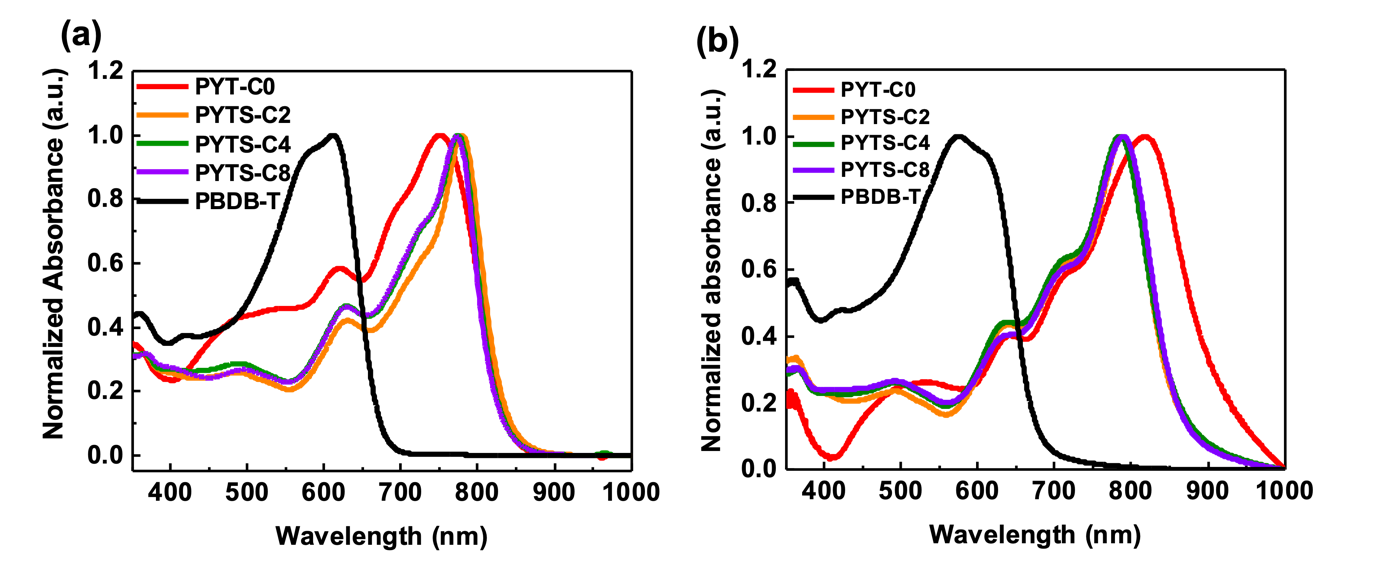
**

**Fig. S4** Normalized absorption spectra of *P*_A_s and PBDB-T in (**a**) CF solutions at room temperature, and (**b**) in pristine films.

**
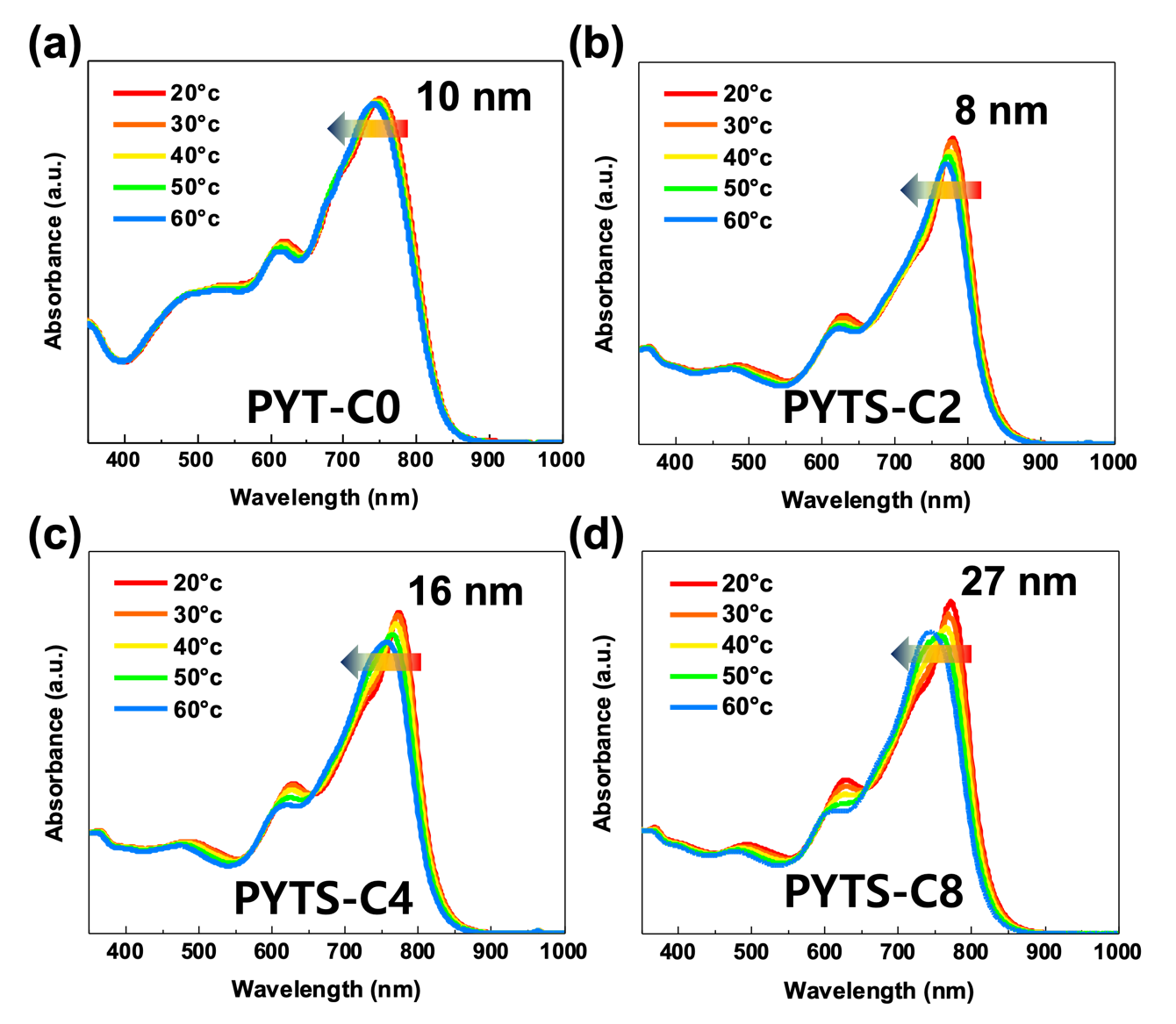
**

**Fig. S5** Temperature-dependent UV-Vis absorption spectra for *P*_A_s in CF at a concentration of 0.02 mg mL^-1^.

**Fig. S6** GIWAXS linecut profiles of the pristine polymers in (**a**) IP and (**b**) OOP directions.

**Table S3** GIWAXS characteristics of the pristine polymers

| **Pristine** | ***d*_100_^IP^**  **[Å]** | ***L*_c,100_^IP^**  **[nm]** | ***d*_010_^OOP^**  **[Å]** | ***L*_c,010_^OOP^**  **[nm]** |
| --- | --- | --- | --- | --- |
| PBDB-T | 22.12 | 6.53 | 3.76 | 1.54 |
| PYT-C0 | 16.85 | 5.04 | 3.71 | 2.32 |
| PYTS-C2 | 16.85 | 0.88 | 3.79 | 1.90 |
| PYTS-C4 | 16.85 | 0.99 | 3.80 | 1.80 |
| PYTS-C8 | 16.85 | 6.05 | 3.78 | 2.14 |

**
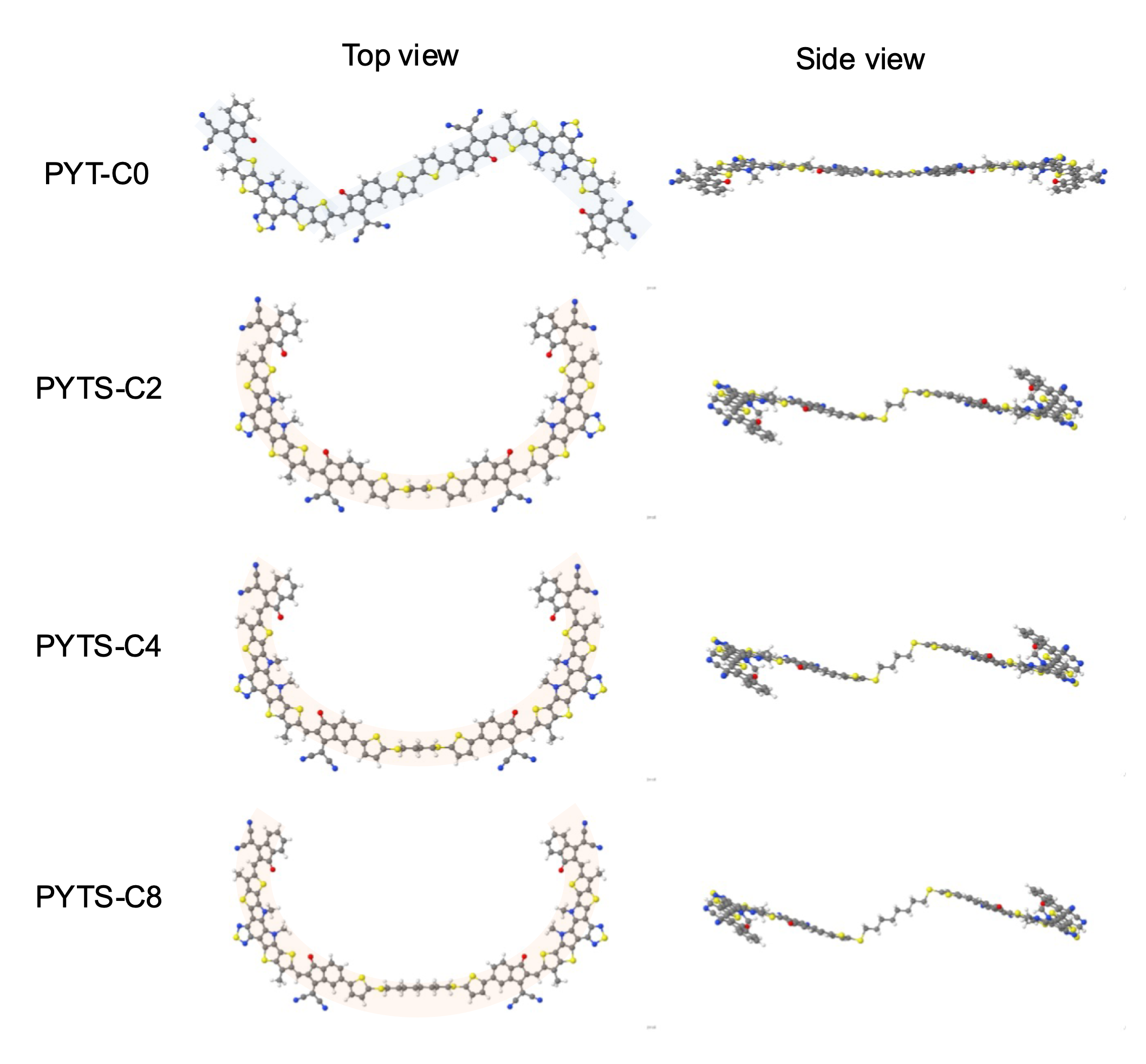
**

**Fig. S7** Vacuum optimized geometries of PYT(S)-Cn (n=0, (without C-S bonds) and 2, 4, and 8 carbon atoms) using TPSSh/6-31G(d). Methyl side chain is applied on YBO core instead of long alkyl side chains for the calculation.


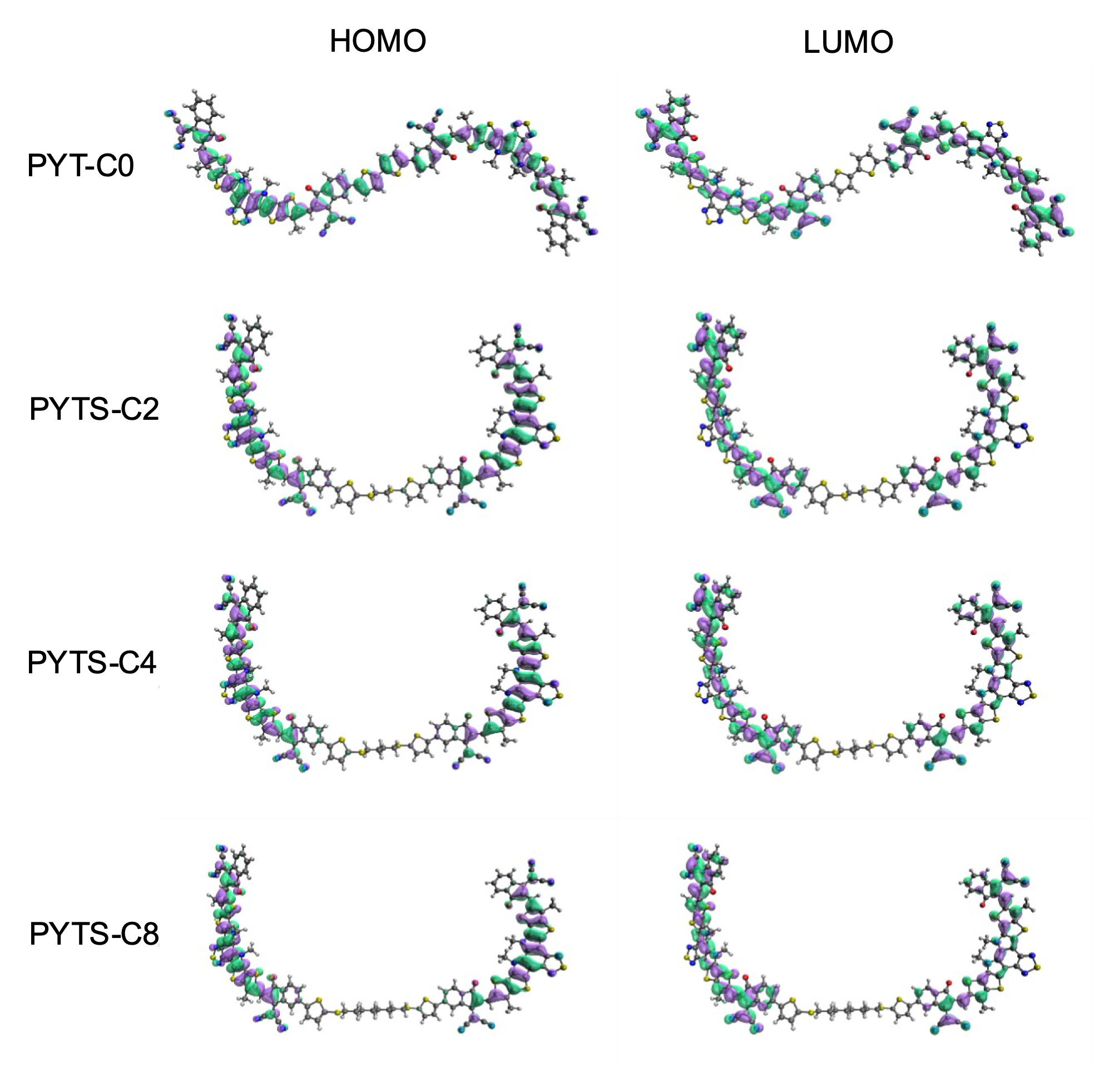


**Fig. S8** HOMO and LUMO orbitals of PYT(S)-Cn (n=0, (without C-S bonds) and 2, 4, and 8 carbon atoms) using TPSS/6-311G(d,p). Orbitals plotted with the program IQmol using an isovalue of 0.04. Methyl side chain is applied on YBO core instead of long alkyl side chains for the calculation.

**Fig. S9** Electronic excitations and absorption spectra of PYT(S)-Cn (n=0 (no C-S bonds either), 2, 4, and 8 carbon atoms) in CF solution. Methyl side chain is applied on YBO core instead of long alkyl side chains for the calculation. Excitations calculated with TD-TPSSh/6-311G(d,p) and absorption spectra obtained from the convolution of Lorentzian functions with 0.1 eV linewidth centered in each electronic excitation energy. Solvent effects treated by the SMD model. Hole and electron pictures for the most intense excitation obtained by computing the Natural Transition Orbitals (NTOs).

**Table S4** The first 5 electronic excitations of PYT(S)-Cn (n=0 (no C-S bonds either), 2, 4, and 8 carbon atoms) calculated with TD-DFT and the absorption maximum obtained from measurements

| From TD-DFT modelling (TPSSh/6-311G(d,p)) in chloroform solution | | | | | | | From measurements | |
| --- | --- | --- | --- | --- | --- | --- | --- | --- |
| System | State | Most important contributions | | E (eV) | λ (nm) | Intensity | λ_MAX_ (nm) Solution at 20 ºC | λ_MAX_ (nm) Film |
| PYT-C0 | 1 | HOMO > LUMO |  | 1.50 | 826.9 | 2.151 | **752** | **818** |
|  | 2 | HOMO > LUMO+1 | HOMO-1 > LUMO | 1.52 | 817.1 | 0.002 |  |  |
|  | 3 | HOMO-1 > LUMO+1 |  | 1.57 | **788.6** | 2.379 |  |  |
|  | 4 | HOMO-1 > LUMO | HOMO > LUMO+1 | 1.59 | 780.6 | 0.012 |  |  |
|  | 5 | HOMO-2 > LUMO | HOMO > LUMO+2 | 1.72 | 722.9 | 0.707 |  |  |
| PYTS-C2 | 1 | HOMO > LUMO | HOMO-1 > LUMO+1 | 1.56 | 792.4 | 0.005 | **779** | **788** |
|  | 2 | HOMO > LUMO+1 | HOMO-1 > LUMO | 1.56 | 792.3 | 0.003 |  |  |
|  | 3 | HOMO-1 > LUMO+1 | HOMO > LUMO | 1.58 | 783.6 | 0.881 |  |  |
|  | 4 | HOMO-1 > LUMO | HOMO > LUMO+1 | 1.60 | **777.2** | 3.724 |  |  |
|  | 5 | HOMO > LUMO+2 | HOMO-1 > LUMO+3 | 1.82 | 680.0 | 0.001 |  |  |
| PYTS-C4 | 1 | HOMO > LUMO | HOMO-1 > LUMO+1 | 1.57 | 791.2 | 0.002 | **774** | **785** |
|  | 2 | HOMO > LUMO+1 | HOMO-1 > LUMO | 1.57 | 791.2 | 0.002 |  |  |
|  | 3 | HOMO-1 > LUMO+1 | HOMO > LUMO | 1.58 | 783.3 | 0.832 |  |  |
|  | 4 | HOMO-1 > LUMO | HOMO > LUMO+1 | 1.59 | **778.3** | 3.760 |  |  |
|  | 5 | HOMO > LUMO+2 | HOMO-1 > LUMO+3 | 1.82 | 679.5 | 0.000 |  |  |
| PYTS-C8 | 1 | HOMO > LUMO | HOMO-1 > LUMO+1 | 1.57 | 788.4 | 0.000 | **772** | **789** |
|  | 2 | HOMO > LUMO+1 | HOMO-1 > LUMO | 1.57 | 788.4 | 0.000 |  |  |
|  | 3 | HOMO-1 > LUMO+1 | HOMO > LUMO | 1.58 | 782.4 | 0.874 |  |  |
|  | 4 | HOMO-1 > LUMO | HOMO > LUMO+1 | 1.59 | **779.3** | 3.723 |  |  |
|  | 5 | HOMO > LUMO+2 | HOMO-1 > LUMO+3 | 1.83 | 677.5 | 0.000 |  |  |


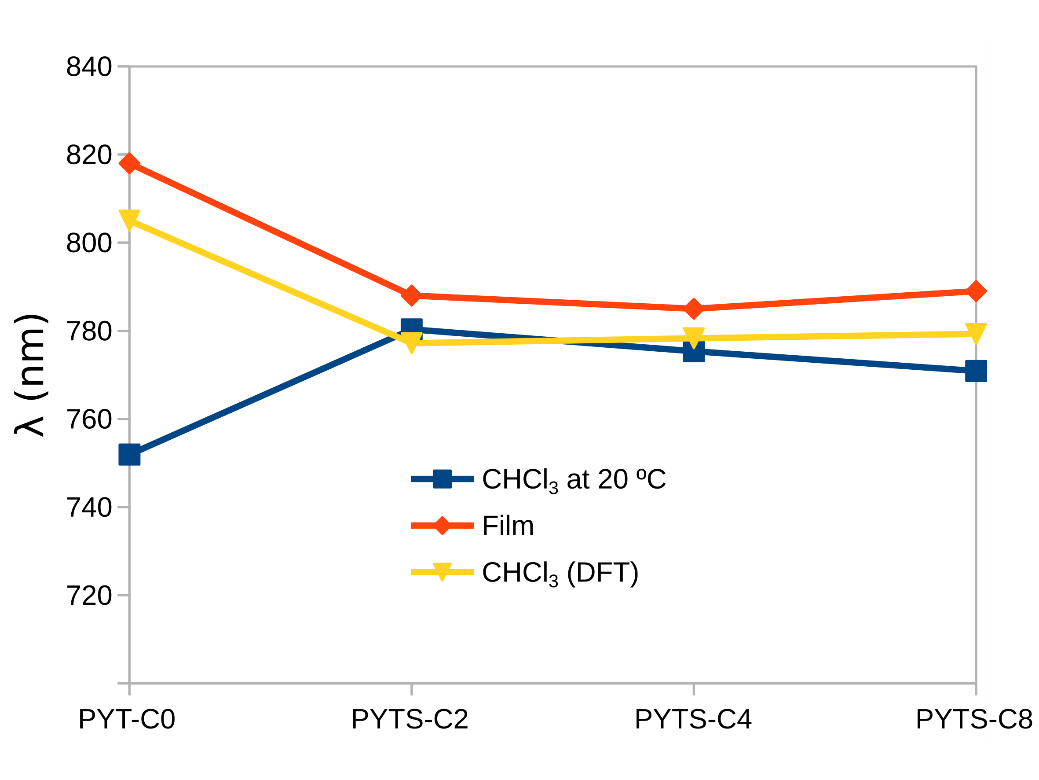


**Fig. S10** Comparison of absorption maximum for PYT(S)-Cn (n=0 (no C-S bonds either), 2, 4, and 8 carbon atoms) in CF at 20 ℃ (blue line), in film (red line) and by the Lorentzian convolution of the TD-TPSSh/6-311G(d,p) excitations (yellow line).

**Fig. S11** *J–V* curves of all-PSCs based on PBDB-T:PYTS-C2 with different molecular weights and PDI of PYTS-C2 (*M*_n_ = 39.9 kg mol^-1^, PDI = 1.77 vs. *M*_n_ = 19.1 kg mol^-1^, PDI = 3.01).

**Table S5** PV performances of the all-PSCs based on PYTS-C2 with different molecular weights

| ***P*_A_** | ***M*_n_**  **[kg mol ^–1^]** | ***PÐI*** | ***V*_oc_**  **[V]** | ***J*_sc_**  **[mA cm^−2^]** | **FF** | **PCE_max_**  **[%]** |
| --- | --- | --- | --- | --- | --- | --- |
| PYTS-C2 | 39.9 | 1.77 | 0.91 | 18.79 | 0.67 | 11.37 |
| PYTS-C2 | 19.1 | 3.01 | 0.90 | 16.80 | 0.60 | 9.11 |

**Table S6** SCLC mobilities of the PBDB-T: *P*_A_s blends

| **Blend**  **[w/ PBDB-T]** | ***μ*_e,blend_**  **[cm^2^ V^-1^ s^-1^]** | ***μ*_h,blend_**  **[cm^2^ V^-1^ s^-1^]** |
| --- | --- | --- |
| PYT-C0 | 2.1 × 10^-6^ | 3.3 × 10^-6^ |
| PYTS-C2 | 1.1 × 10^-5^ | 4.2 × 10^-5^ |
| PYTS-C4 | 1.5 × 10^-6^ | 2.1 × 10^-5^ |
| PYTS-C8 | 8.0 × 10^-6^ | 3.0 × 10^-5^ |


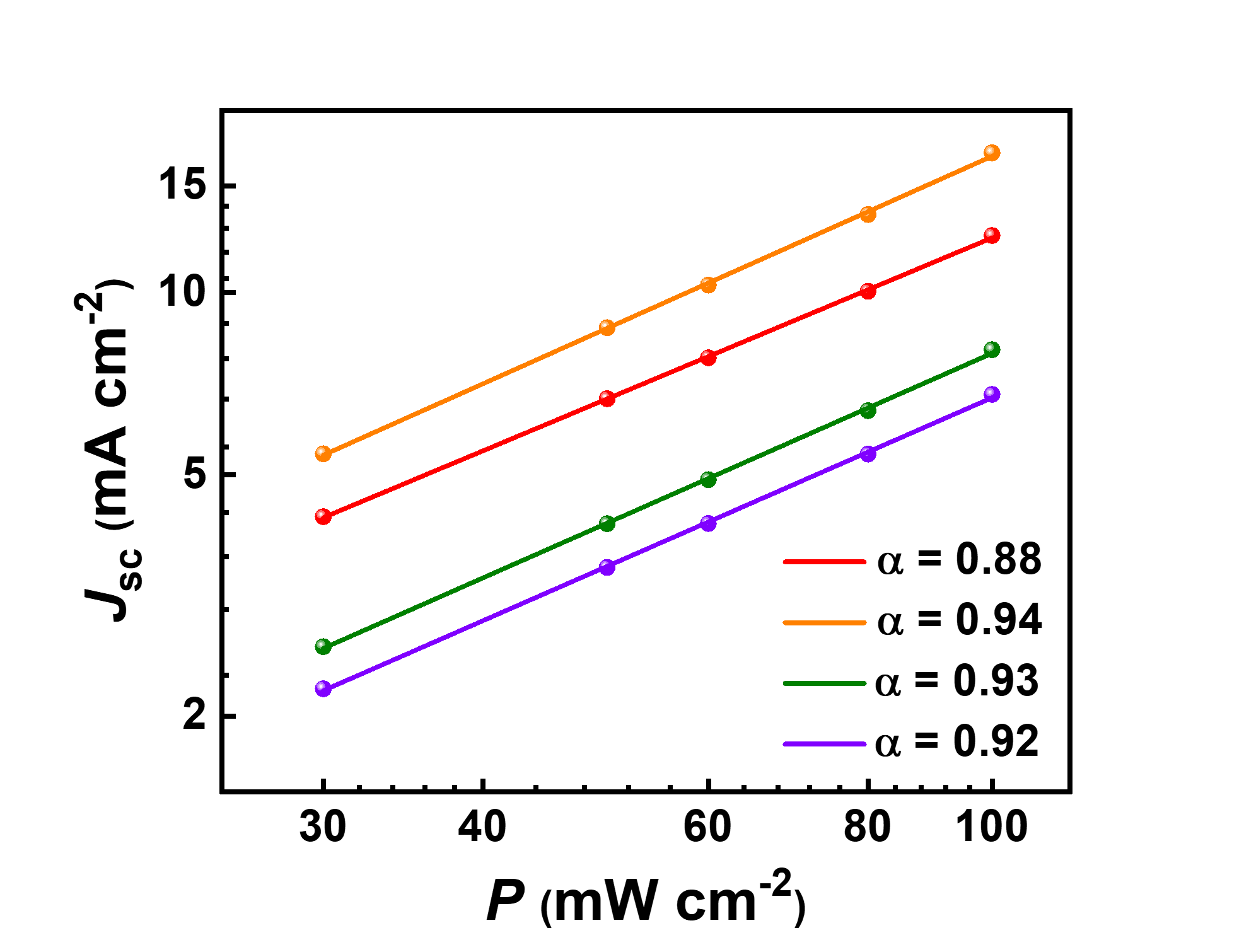


**Fig. S12** Dependences of *J*_sc_ on light intensities in all-PSCs.

**Fig. S13** *Stress–Strain* curves for the pristine PYTS-C2 film.

**Table S7** Mechanical properties of the PYTS-C2 film measured from the pseudo free-standing tensile test

| **Blend** | ***E***  **[Mpa]*^a^*** | **COS**  **[%]*^a^*** | **Toughness**  **[MJ m^–3^]*^a^*** |
| --- | --- | --- | --- |
| PYTS-C2 | 880.14 ± 141.17 | 2.81 ± 0.22 | 0.34 ± 0.05 |

*^a^* All parameters represent average values from 3 samples.

**
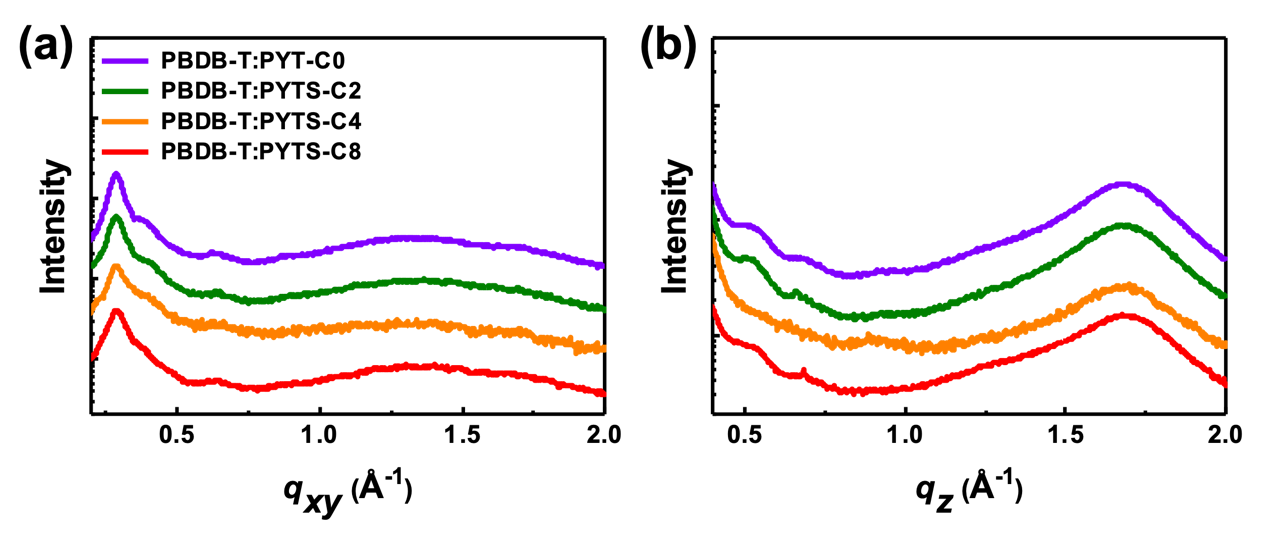
**

**Fig. S14** GIWAXS linecut profiles of the PBDB-T:*P*_A_s blends in (**a**) IP and (**b**) OOP directions.

**Table S8** GIWAXS characteristics of the PBDB-T:*P*_A_s blends

| **Blend** | ***d*_100_^IP^**  **(Å)** | ***L*_c,100_^IP^**  **(nm)** | ***d*_010_^OOP^**  **(Å)** | ***L*_c,010_^OOP^**  **(nm)** |
| --- | --- | --- | --- | --- |
| PYT-C0 | 21.59 | 11.32 | 3.74 | 2.98 |
| PYTS-C2 | 21.59 | 10.39 | 3.73 | 2.02 |
| PYTS-C4 | 21.59 | 11.09 | 3.75 | 2.49 |
| PYTS-C8 | 21.67 | 13.04 | 3.74 | 2.84 |

**Table S9** Contact angle data of PBDB-T and *P*_A_s

|  | **Contact angle**  **[°]** | | **Surface tension**  **[mN m^-1^]*^a^*** | **Interfacial tension**  **[mN m^-1^]*^a^*** |
| --- | --- | --- | --- | --- |
|  | **Water** | **Glycerol** |  |  |
| PBDB-T | 102.7 | 88.4 | 23.27 | - |
| PYT-C0 | 85.6 | 77.9 | 27.85 | 6.31 |
| PYTS-C2 | 91.8 | 78.1 | 27.76 | 1.38 |
| PYTS-C4 | 92.0 | 78.5 | 27.48 | 1.44 |
| PYTS-C8 | 91.6 | 81.1 | 25.75 | 2.82 |

*^a^* The interfacial tensions were calculated using Wu model [9-11].

**Nuclear Magnetic Resonance Spectra:**


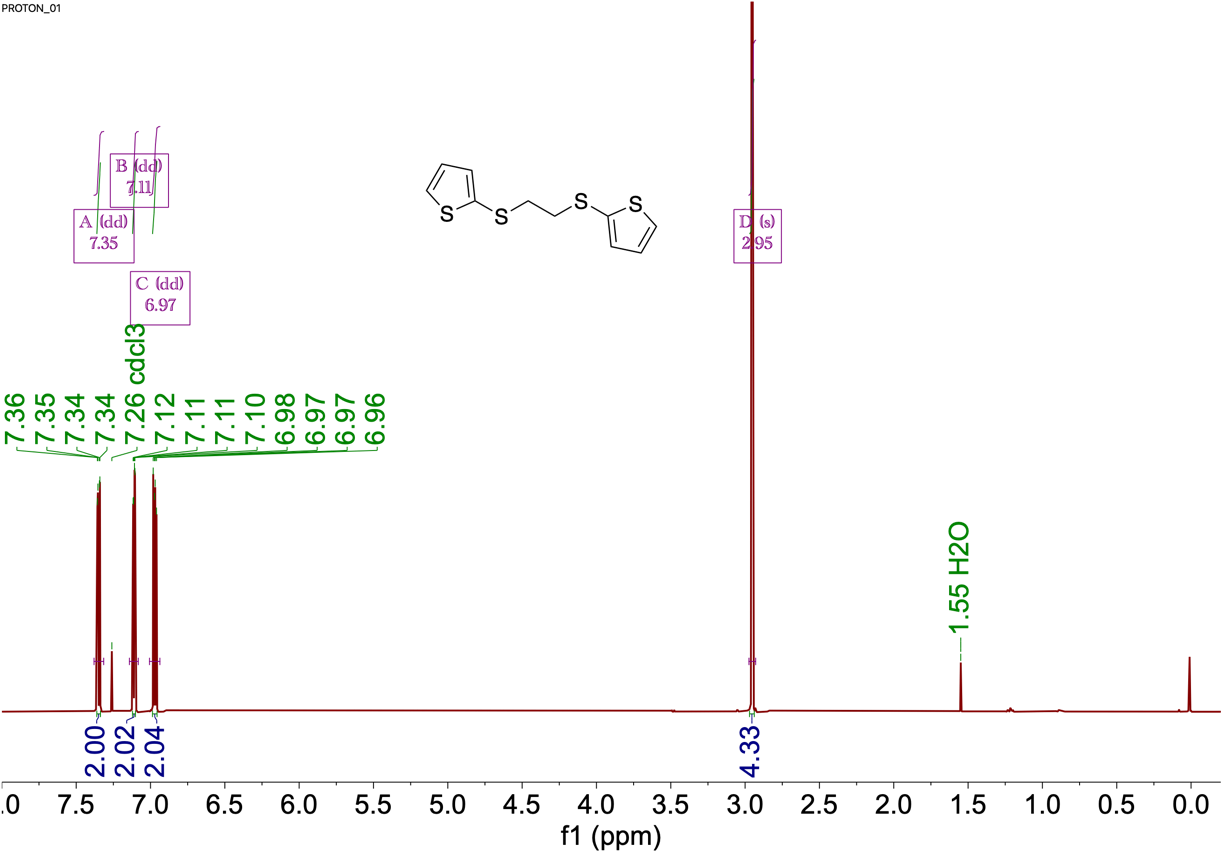


**Fig. S15** ^1^H NMR spectrum (400 MHz) for **TS-C2** taken in CDCl_3_ at 25 ℃.

**
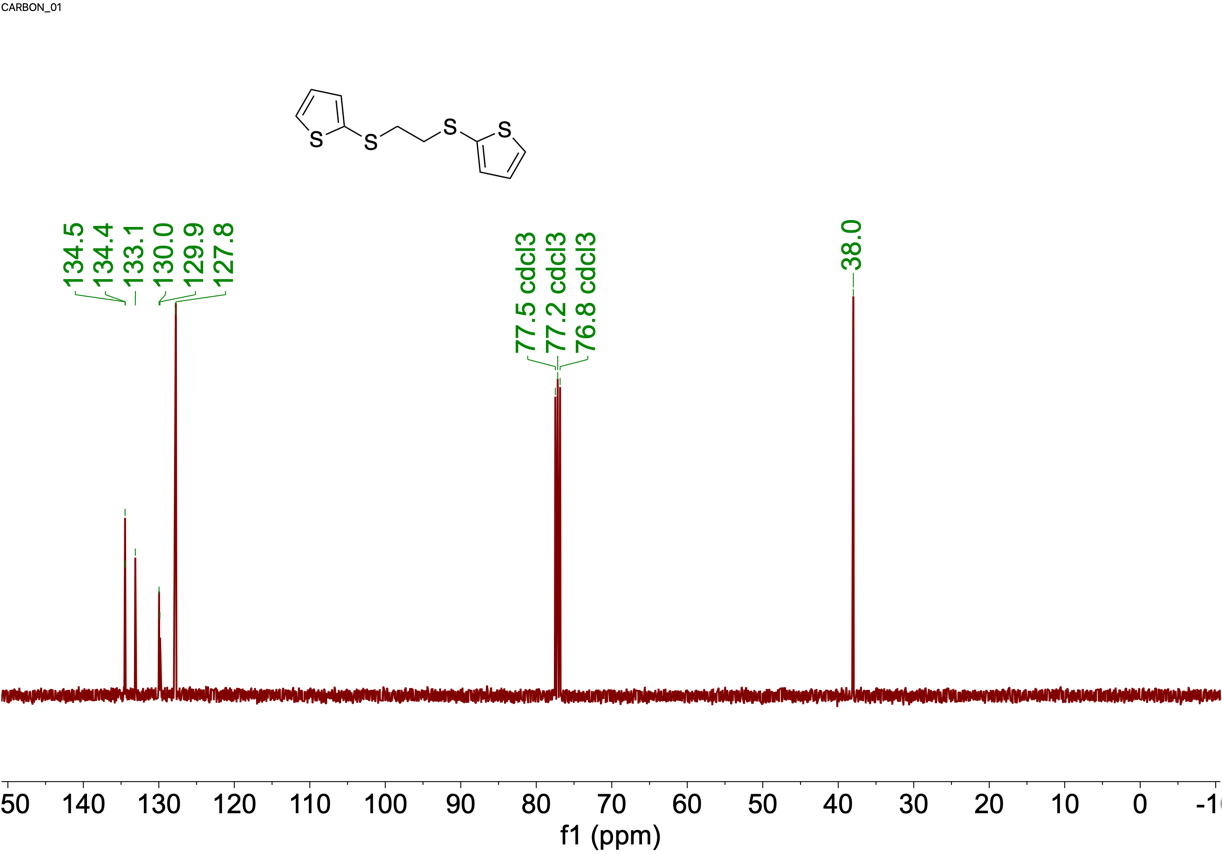
**

**Fig. S16** ^13^C NMR spectrum (100 MHz) for **TS-C2** taken in CDCl_3_ at 25 ℃.


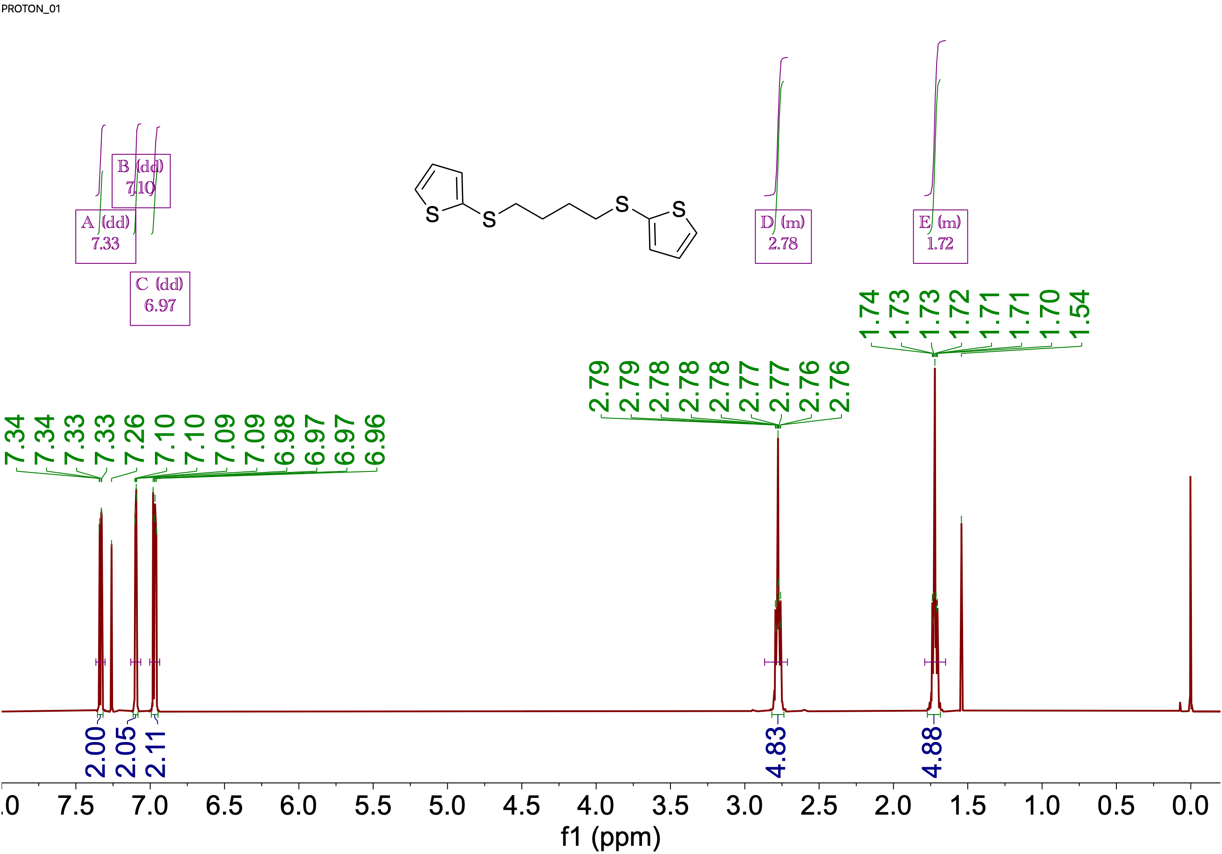


**Fig. S17** ^1^H NMR spectrum (400 MHz) for **TS-C4** taken in CDCl_3_ at 25 ℃.

**
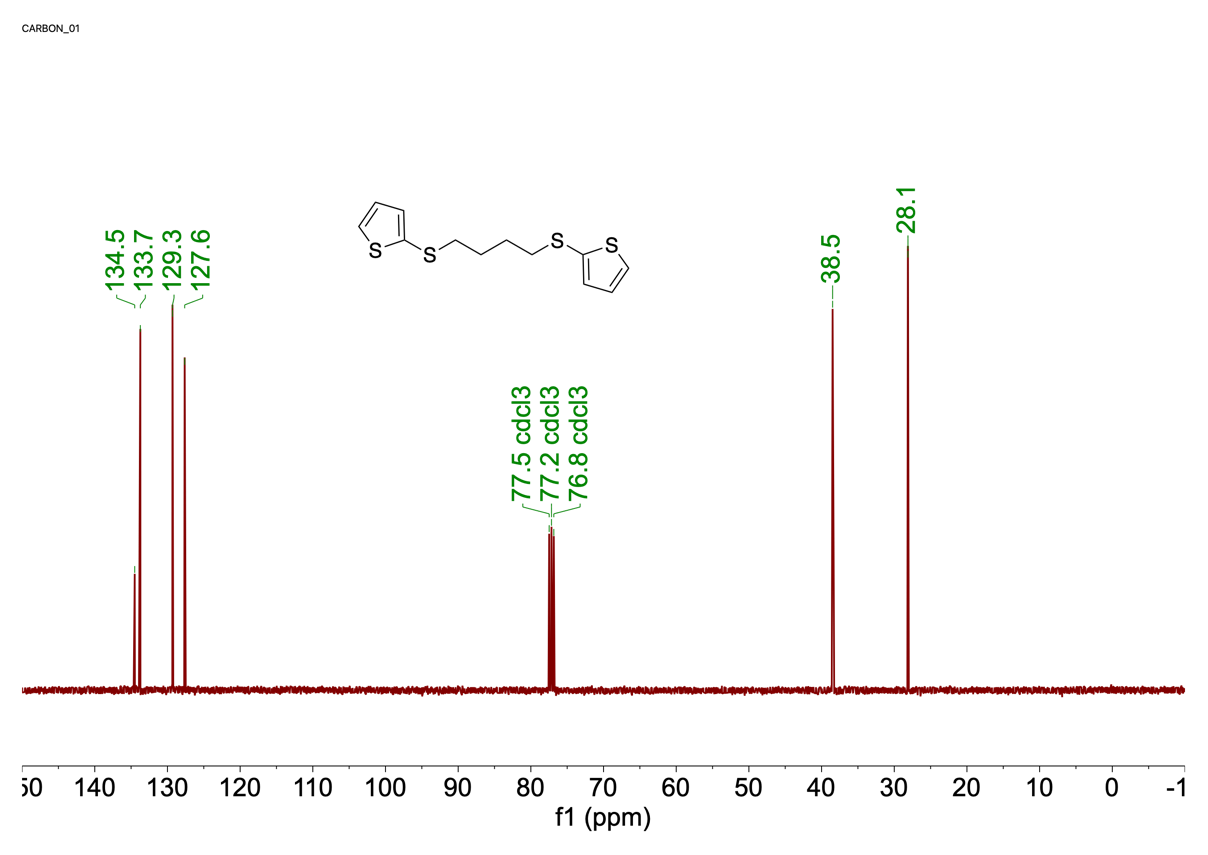
**

**Fig. S18** ^13^C NMR spectrum (100 MHz) for **TS-C4** taken in CDCl_3_ at 25 ℃.


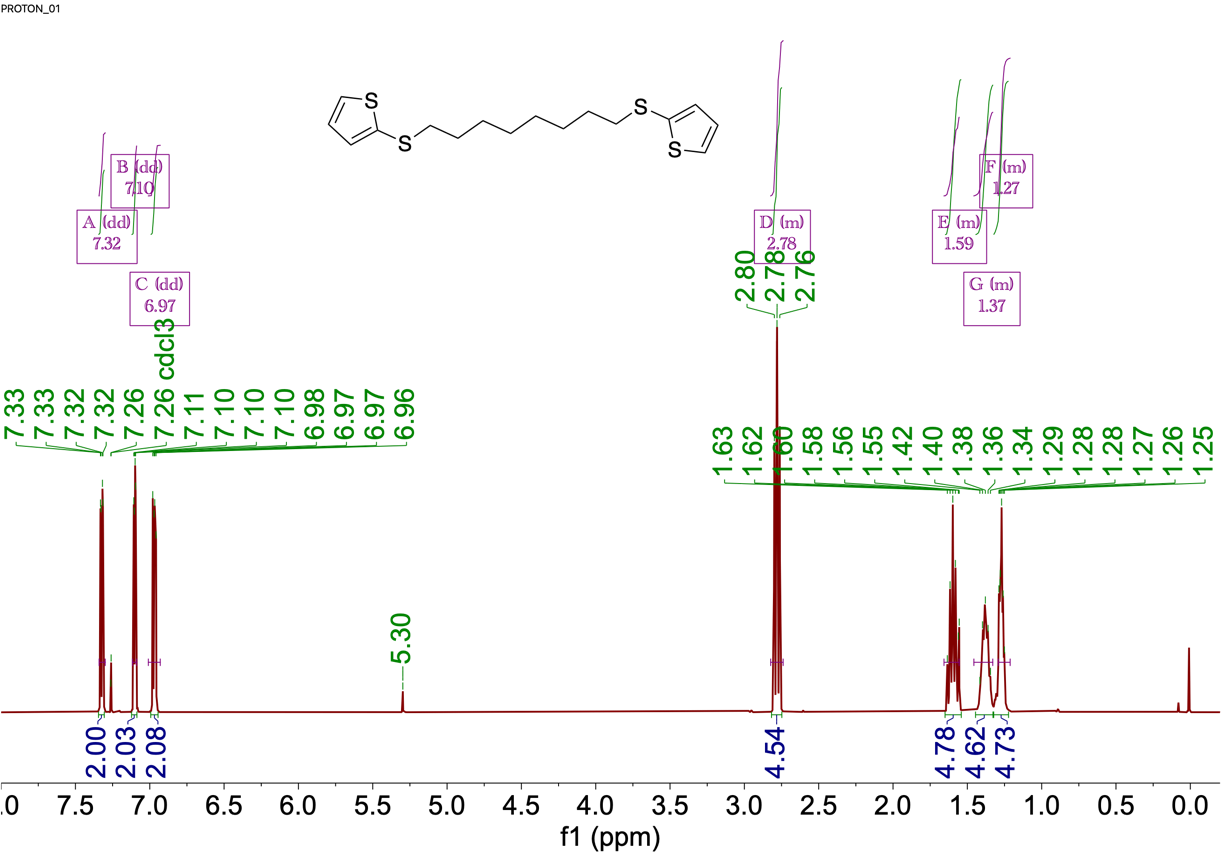


**Fig. S19** ^1^H NMR spectrum (400 MHz) for **TS-C8** taken in CDCl_3_ at 25 ℃.

**
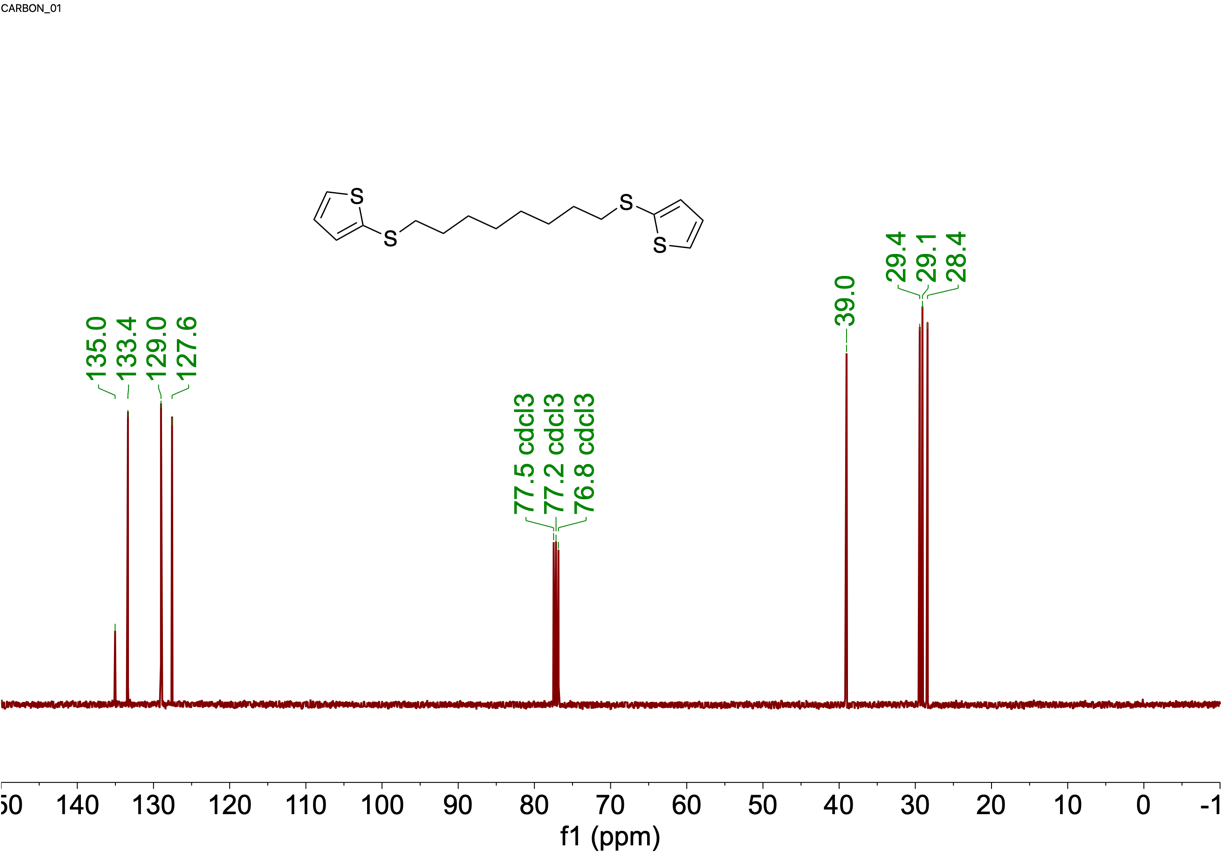
**

**Fig. S20** ^13^C NMR spectrum (100 MHz) for **TS-C8** taken in CDCl_3_ at 25 ℃.


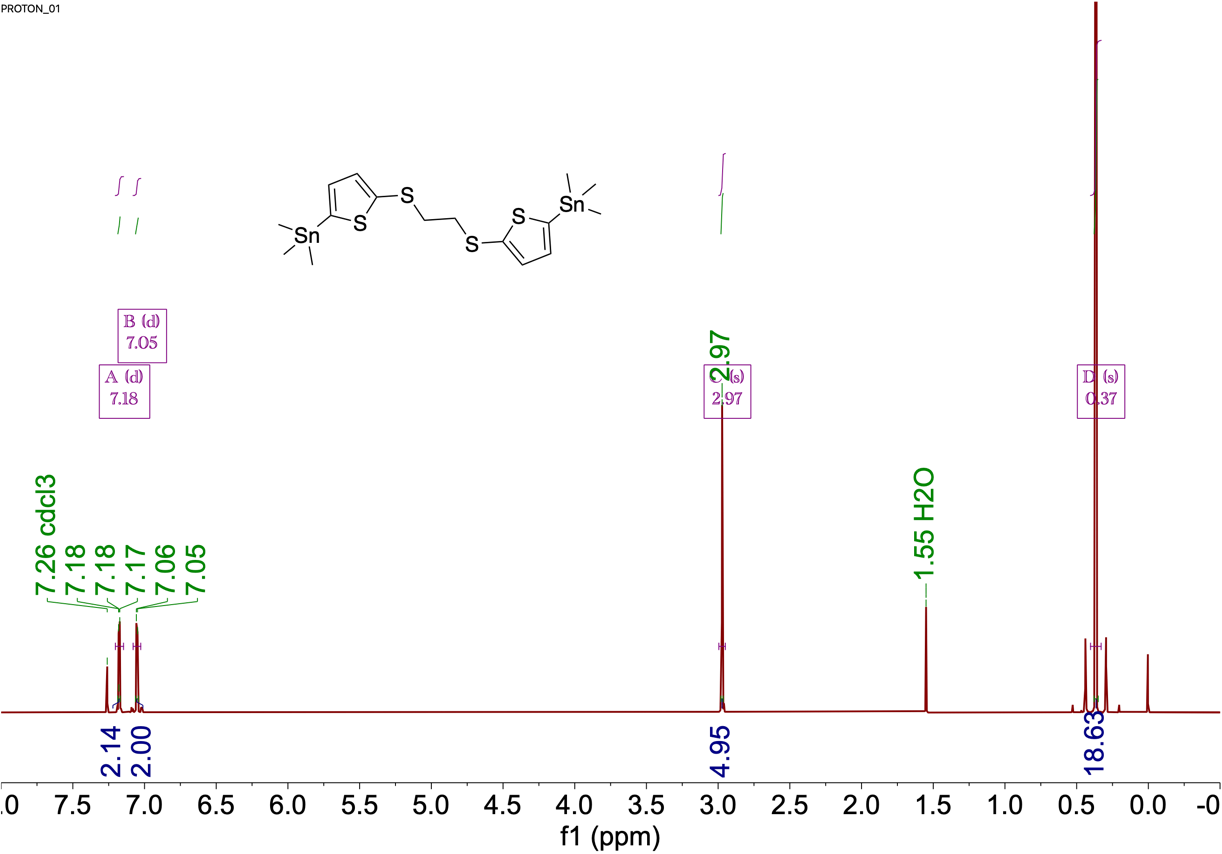


**Fig. S21** ^1^H NMR spectrum (400 MHz) for **TS-C2-Sn** taken in CDCl_3_ at 25 ℃.

**
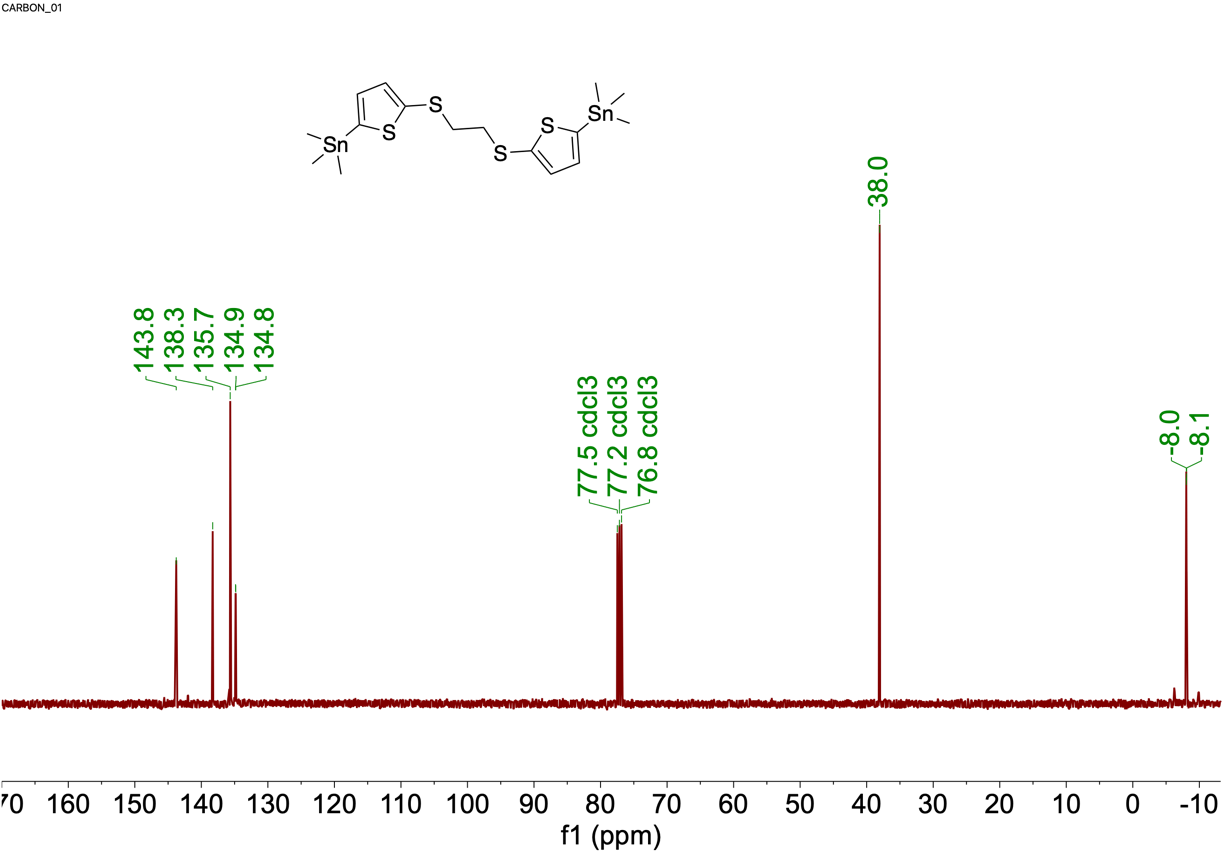
**

**Fig. S22** ^13^C NMR spectrum (100 MHz) for **TS-C2-Sn** taken in CDCl_3_ at 25 ℃.


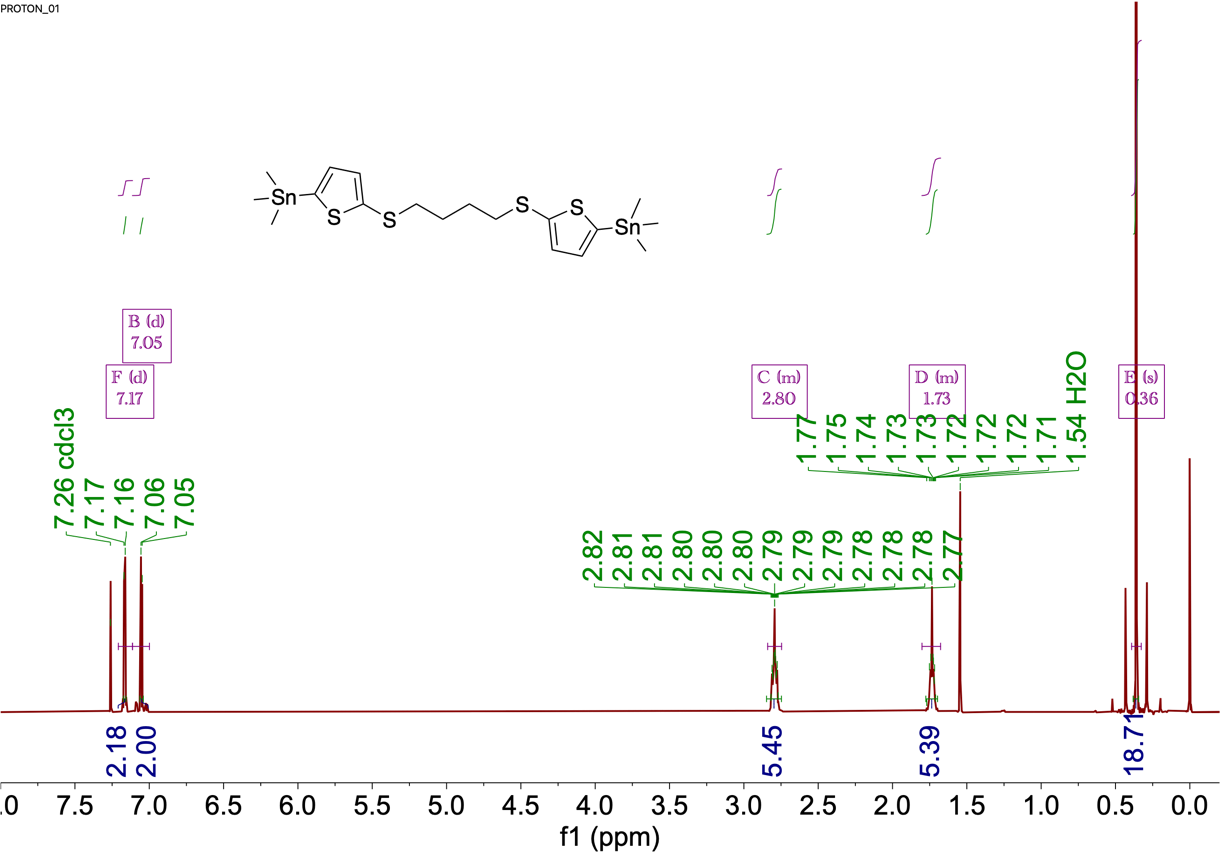


**Fig. S23** ^1^H NMR spectrum (400 MHz) for **TS-C4-Sn** taken in CDCl_3_ at 25 ℃.

**
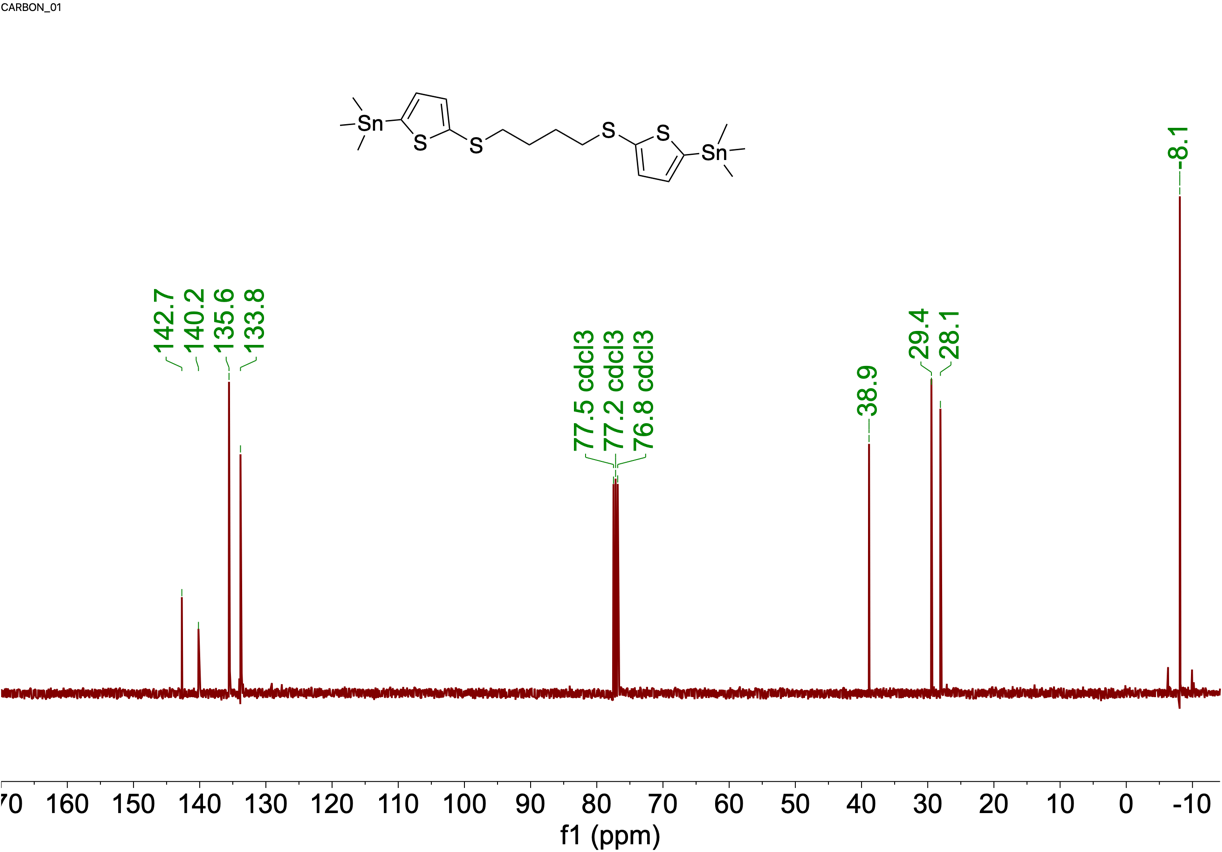
**

**Fig. S24** ^13^C NMR spectrum (100 MHz) for **TS-C4-Sn** taken in CDCl_3_ at 25 ℃.


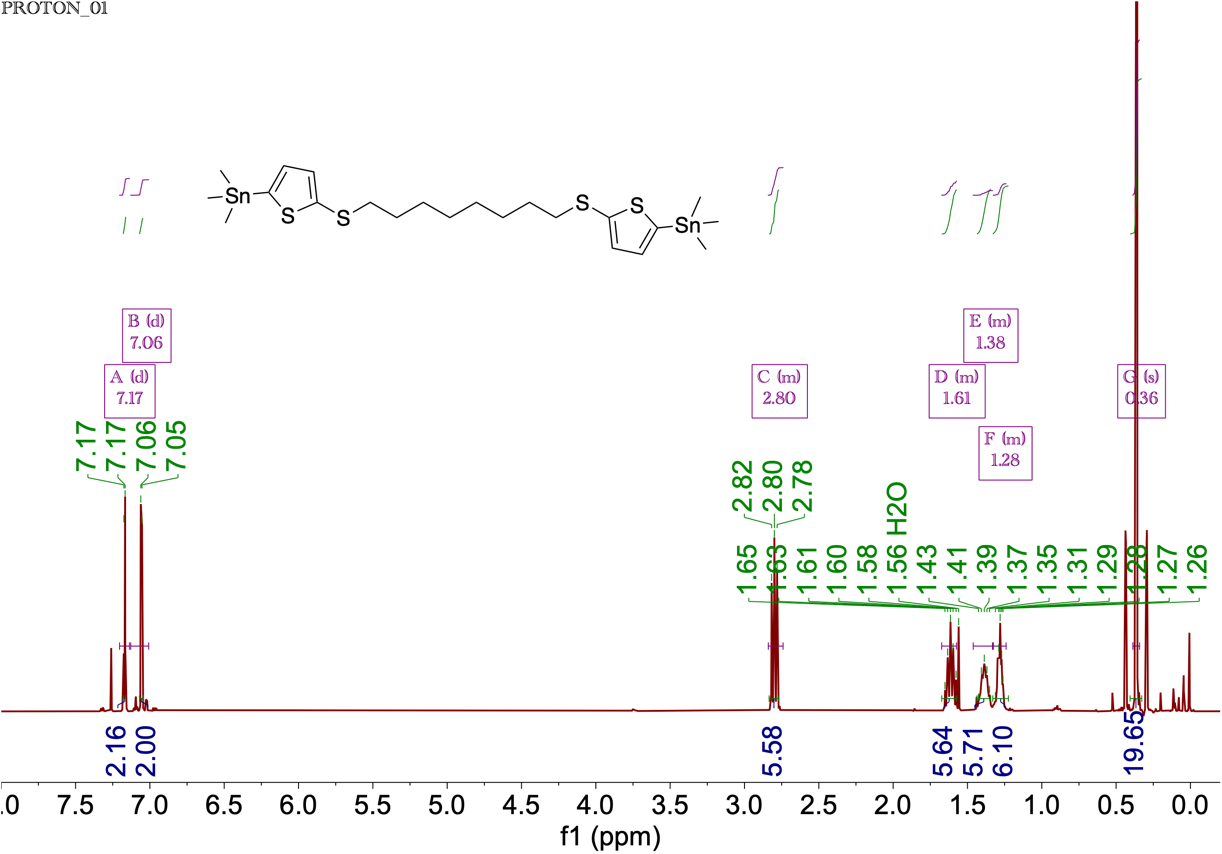


**Fig. S25** ^1^H NMR spectrum (400 MHz) for **TS-C8-Sn** taken in CDCl_3_ at 25 ℃.

**
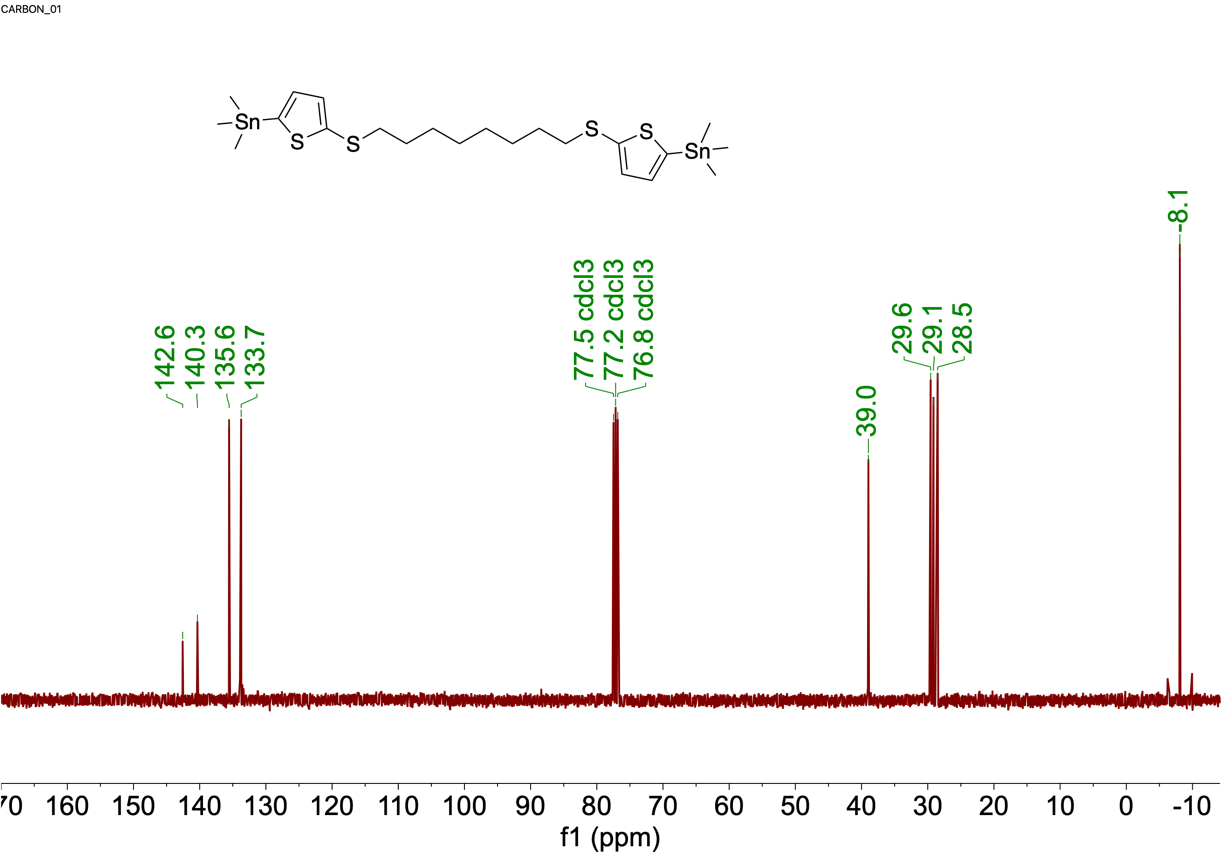
**

**Fig. S26** ^13^C NMR spectrum (100 MHz) for **TS-C8-Sn** taken in CDCl_3_ at 25 ℃.

**Supplemental References**

[S1] Z. Genene, J.-W. Lee, S.-W. Lee, Q. Chen, Z. Tan, B. A. Abdulahi, D. Yu, T.-S. Kim, B. J. Kim, E. Wang. Polymer acceptors with flexible spacers afford efficient and mechanically robust all-polymer solar cells. Adv. Mater. **34**(6), 2107361(2020). https://doi.org/10.1002/adma.202107361

[S2] V. V. Pavlishchuk, A. W. Addison. Conversion constants for redox potentials measured versus different reference electrodes in acetonitrile solutions at 25 C. Inorganica Chim. Acta **298**(1), 97-102 (2000). <https://doi.org/10.1016/S0020-1693(99)00407-7>

[S3] A. J. Bard., L. R. Faulkner, Student Solutions Manual to accompany Electrochemical Methods: Fundamentals and Applicaitons, 2e. (John Wiley and Sons; 2002), pp.

[S4] R. Ditchfield, W. J. Hehre, J. A. Pople. Self‐consistent molecular‐orbital methods. Ix. An extended gaussian‐type basis for molecular‐orbital studies of organic molecules. J. Chem. Phys. **54**(2), 724-728 (1971). <https://doi.org/10.1063/1.1674902>

[S5] M. S. Gordon, J. S. Binkley, J. A. Pople, W. J. Pietro, W. J. Hehre. Self-consistent molecular-orbital methods. 22. Small split-valence basis sets for second-row elements. J. Am. Chem. Soc. **104**(10), 2797-2803 (1982). <https://doi.org/10.1021/ja00374a017>

[S6] A. V. Marenich, C. J. Cramer, D. G. Truhlar. Universal solvation model based on solute electron density and on a continuum model of the solvent defined by the bulk dielectric constant and atomic surface tensions. J. Phys. Chem. B. **113**(18), 6378-6396 (2009). https://doi.org/10.1021/jp810292n

[S7] M. Frisch, G. Trucks, H. Schlegel, G. Scuseria, M. Robb, J. Cheeseman, G. Scalmani, V. Barone, G. Petersson, H. Nakatsuji. Gaussian 16. (2016)

[S8] Q. Fan, R. Ma, T. Liu, J. Yu, Y. Xiao, W. Su, G. Cai, Y. Li, W. Peng, T. Guo, Z. Luo, H. Sun, L. Hou, W. Zhu, X. Lu, F. Gao, E. Moons, D. Yu, H. Yan, E. Wang. High-performance all-polymer solar cells enabled by a novel low bandgap non-fully conjugated polymer acceptor. Sci. China Chem. **64**(8), 1380-1388 (2021). https://doi.org/10.1007/s11426-021-1020-7

[S9] H. Kang, K.-H. Kim, J. Choi, C. Lee, B. J. Kim. High-performance all-polymer solar cells based on face-on stacked polymer blends with low interfacial tension. Acs Macro. Lett. **3**(10), 1009-1014 (2014). https://doi.org/10.1021/mz500415a

[S10] J.-W. Lee, C. Sun, B. S. Ma, H. J. Kim, C. Wang, J. M. Ryu, C. Lim, T. S. Kim, Y.-H. Kim, S.-K. Kwon, B. J. Kim. Efficient, thermally stable, and mechanically robust all‐polymer solar cells consisting of the same benzodithiophene unit‐based polymer acceptor and donor with high molecular compatibility. Adv. Energy Mater. **11**(5), 2003367 (2021). https://doi.org/10.1002/aenm.202003367

[S11] S. Wu. Calculation of interfacial tension in polymer systems. J. Polym. Sci. Part C Polym. Symp. **34**(1), 19-30 (1971). https://doi.org/10.1002/polc.5070340105
